# Leg length and bristle density, both necessary for water surface locomotion, are genetically correlated in water striders

**DOI:** 10.1073/pnas.2119210119

**Published:** 2022-02-22

**Authors:** Cédric Finet, Amélie Decaras, Maria Rutkowska, Pascale Roux, Samuel Collaudin, Pauline Joncour, Séverine Viala, Abderrahman Khila

**Affiliations:** ^a^Institut de Génomique Fonctionnelle de Lyon, Univ Lyon, CNRS UMR 5242, INRAE USC 1370 École Normale Supérieure de Lyon, Université Claude Bernard Lyon1, Lyon 69364, France

**Keywords:** correlated traits, evolution and adaptation, pleiotropy, cell division, water striders

## Abstract

Access to hitherto unexploited ecological opportunities is associated with phenotypic evolution and often results in significant lineage diversification. Yet our understanding of the mechanisms underlying such adaptive traits remains limited. Water striders have been able to exploit the water–air interface, primarily facilitated by changes in the density of hydrophobic bristles and a significant increase in leg length. These two traits are functionally correlated and are both necessary for generating efficient locomotion on the water surface. Whether bristle density and leg length have any cellular or developmental genetic mechanisms in common is unknown. Here, we combine comparative genomics and transcriptomics with functional RNA interference assays to examine the developmental genetic and cellular mechanisms underlying the patterning of the bristles and the legs in *Gerris buenoi* and *Mesovelia mulsanti*, two species of water striders. We found that two duplication events in the genes *beadex* and *taxi* led to a functional expansion of the paralogs, which affected bristle density and leg length. We also identified genes for which no function in bristle development has been previously described in other insects. Interestingly, most of these genes play a dual role in regulating bristle development and leg length. In addition, these genes play a role in regulating cell division. This result suggests that cell division may be a common mechanism through which these genes can simultaneously regulate leg length and bristle density. We propose that pleiotropy, through which gene function affects the development of multiple traits, may play a prominent role in facilitating access to unexploited ecological opportunities and species diversification.

## Significance

When organisms access unexploited ecological opportunities, species diversification often follows, yet the mechanisms underlying such transitions are poorly understood. Water striders transited from terrestrial to water surface life some 200 Mya, aided by the evolution of superhydrophobic bristles and increased leg length, both required for standing and moving on water. We identified genes involved in both bristle density and leg length, suggesting that these two traits are genetically correlated. Strikingly, these genes are involved in cell division, thus explaining their dual role in leg growth and bristle density. In addition, we identified molecular changes that account in part for the differences in bristle density across species. We propose that pleiotropy might facilitate diversification by impacting several adaptive traits simultaneously.

Access to previously unexploited ecological opportunities is associated with phenotypic evolution and often results in significant lineage diversification. The semiaquatic bugs (Heteroptera: Gerromorpha) transited to life on the surface of various water bodies such as puddles, streams, lakes, mangroves, and even oceans (1–3). Their ancestor was inferred to be a terrestrial bug that evolved the ability to stand and move on the water–air interface around 200 Mya (4). Early-diverging lineages occupy transitional zones and walk both on land and water, whereas derived lineages evolved rowing as a novel mode of locomotion on open-water surface (1, 5). Water surface invasion is commonly viewed as a stepwise process that involved both the diversification of leg morphologies and the evolution of water-repellent hairs that allow the insects to exploit surface tension (4, 6, 7).

In particular, water striders evolved two adaptive traits that are essential for water surface locomotion. The first is a significant increase in leg length, which is positively correlated with increased speed on the water (5, 8, 9). The second is the evolution of legs that are superhydrophobic [i.e., extremely difficult to wet (6)]. In the Cassie–Baxter state, liquids can sit upon hierarchical nanostructures, resulting in air pockets that are trapped between the surface and the liquid. The nanogrooved, oriented hairs on water striders’ legs are thought to act as such structures, which may explain the overall superhydrophobicity in conjunction with the cuticle wax on the leg surface (10). Furthermore, the spatial arrangement of these hairs follows an optimized geometry that allows the legs to achieve maximum, load-carrying capacity (11). A recent study integrates hydrophobicity, geometry, and flexibility to explain self-removal of condensed water on the legs of *Gerris remigis* (12). In addition, the semiaquatic bugs maintain the anti-wetting properties of the hair architecture through meticulous care using a set of grooming combs consisting of rows of stiff bristles that are present on the distal tibiae of one, two, and sometimes all three leg pairs along the body.

The hairs covering the legs of water striders have been recently described as innervated bristles (13). Like *Drosophila* (14–16) and *Tribolium* (17), the development of bristle precursors in the Gerromorpha requires the expression of the proneural gene *achaete-scute homolog* (*ASH*) (13). *ASH* RNA interference (RNAi) caused a drastic decrease in the overall number of leg bristles and eliminated tarsal grooming combs (modified bristles) (13). This result suggested the existence of possible genetic correlation in two characters required for water surface locomotion, namely the hydrophobic bristles and the grooming combs. To date, the work on *ASH* constitutes the only study that tackles the molecular mechanisms underlying the development of leg bristles in the Gerromorpha.

Here, we investigate the genetic basis of bristle patterning, bristle density, and bristle planar distribution in water striders, as well as the variability in leg bristle density among gerromorphan species. First, we characterized bristle formation during both embryonic and postembryonic stages at the tissue and cell levels. Then, we investigated bristle development at the genetic level by combining two main approaches: 1) compilation of candidate genes involved in bristle development in *Drosophila* and the search for evolutionary molecular events, such as gene duplications, that could have affected these genes in the Gerromorpha and 2) identification of genes that are differentially expressed between stages and/or species with significant differences in bristle density.

Our results show that an increase in both bristle density and leg length correlates with an increase in cell density. An RNAi screen identified numerous genes involved in regulating cell division that modulate leg length and bristle density, thus implicating cell division as a common mechanism for the development and evolution of two key traits that are essential for water surface locomotion. We propose that pleiotropy plays a more important role than previously thought in water surface invasion and the diversification of Gerromorpha by impacting several adaptive traits simultaneously.

## Results

### Tarsal Hair Density Is Higher in the Gerromorpha.

Previous observations have shown that a dense layer of hydrophobic hairs covers the legs of water striders (4, 7, 18, 19). However, how the density of bristles has evolved in association with the adaptation of the Gerromorpha to water surface locomotion remains untested. We therefore quantified bristle density on the ventral side of midleg tarsi in 10 species encompassing both terrestrial and semiaquatic Heteroptera. Terrestrial bugs have a significantly lower bristle density compared to bugs that live on the water surface (Kruskal–Wallis: K = 83.5, *P* value < 2.2e-16) (*SI Appendix*, Fig. S1). Within the Gerromorpha, early-diverging lineages that walk both on land and water have a bristle density lower than derived lineages specialized in open-water surface (Kruskal–Wallis: K = 83.5, *P* value < 2.2e-16) (*SI Appendix*, Fig. S1). The tarsal bristle density is remarkably low in *Mesovelia mulsanti* (d_mean_ = 7,053 bristles/mm^2^), whereas it is up to twice as high in gerrids such as *Gerris buenoi* (d_mean_ = 1,1370 bristles/mm^2^) or *Limnoporus dissortis* (d_mean_ = 1,3482 bristles/mm^2^).

Because the genome of *G. buenoi* is fully sequenced (20) and gene silencing is effective in this species (8), our analyses will focus primarily on this species as a representative of high-bristle density. For comparison, we use *M. mulsanti*, which belongs to a lineage of water surface–dwelling insects that have the lowest known bristle density. *G. buenoi* and *M. mulsanti* only diverged after the transition to life on the water surface, thus making the comparison more informative than with a terrestrial species that diverged from the Gerromorpha much earlier.

### Relationship between Cell Division and Increased Bristle Density during Postembryonic Development.

Because bristles represent the primary structures that allow the Gerromorpha to exploit surface tension, it is hypothesized that bristle density correlates with body mass (21). The rationale behind this hypothesis is that bristle-dense surfaces would entrap more microscopic air bubbles that would prevent heavier bodies from breaking surface tension. Gerromorpha that branched earlier have primarily small body size, whereas more derived lineages are larger (5). In terms of behavior, specialized species sustain frequent movement on the open waters, while versatile species spend most of the time static of water lilies and other aquatic plants.

**Fig. 1.**
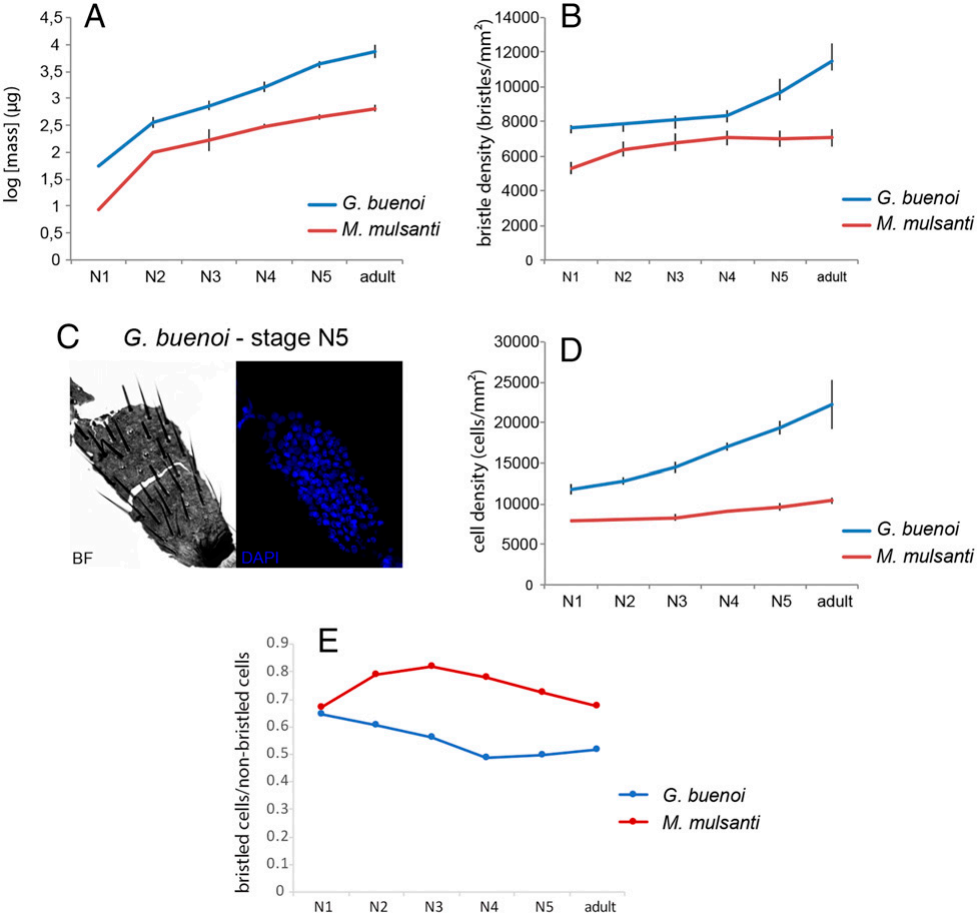
Dynamics of body mass, tarsal bristle density, and tarsal cell density during postembryonic development in *G. buenoi* and *M. mulsanti*. Body mass (*A*), bristle density on the tarsus of leg L2 (*B*), example of DAPI staining used for estimating the number of cells (*C*), cell density on the tarsus of leg L2 (*D*), and ratio number of bristled cells/number of nonbristled cells (*E*). Error bars represent SD. BF: bright field.

By analyzing postembryonic stages, we found that *G. buenoi* first-instar nymphs hatch with significantly higher body weight and bristle density on the tarsi of the midlegs (L2) compared to *M. mulsanti* first-instar nymphs (Fig. 1 *A* and *B*). The bristle density increases, as body weight also increases, between nymphal instar N1 and the adult in the two species, meaning that new bristles are added throughout the molts. The increase in bristle density is linear until nymphal instar N3 in both species but reaches a plateau at stage N4 in *M. mulsanti*, while it keeps increasing during stages N4 and N5 in *G. buenoi* (Fig. 1*B*). This result suggests that the higher-bristle density in *G. buenoi* could be explained by the higher body mass.

This result also raises the question of the cellular and molecular mechanisms underlying the differences between *G. buenoi* and *M. mulsanti*. Analyses of the growth of embryonic legs in the gerrid *L. dissortis* (*SI Appendix*, Fig. S2 *A* and *B*) revealed that leg elongation during embryogenesis (*SI Appendix*, Fig. S2*C*) is associated with an increase in cell number (*SI Appendix*, Fig. S2*D*), which results from a burst of cell division that occurs during midembryogenesis (*SI Appendix*, Fig. S2*E*). We therefore counted DAPI-labeled nuclei in fixed L2 tarsi (Fig. 1*C*) in *G. buenoi* and *M. mulsanti* and found that tarsal cell density increases more significantly in *G. buenoi* compared to *M. mulsanti* over postembryonic development (Fig. 1*D*). Interestingly, the ratio of bristled cells to nonbristled cells is higher in *M. mulsanti* at any given stage (Fig. 1*E*). These results suggest that the low-tarsal bristle density in *M. mulsanti* is constrained by the lower total number of cells.

### Evidence of Functional Change following Duplication in Two-Bristle Development Genes in the Gerromorpha.

**Fig. 2.**
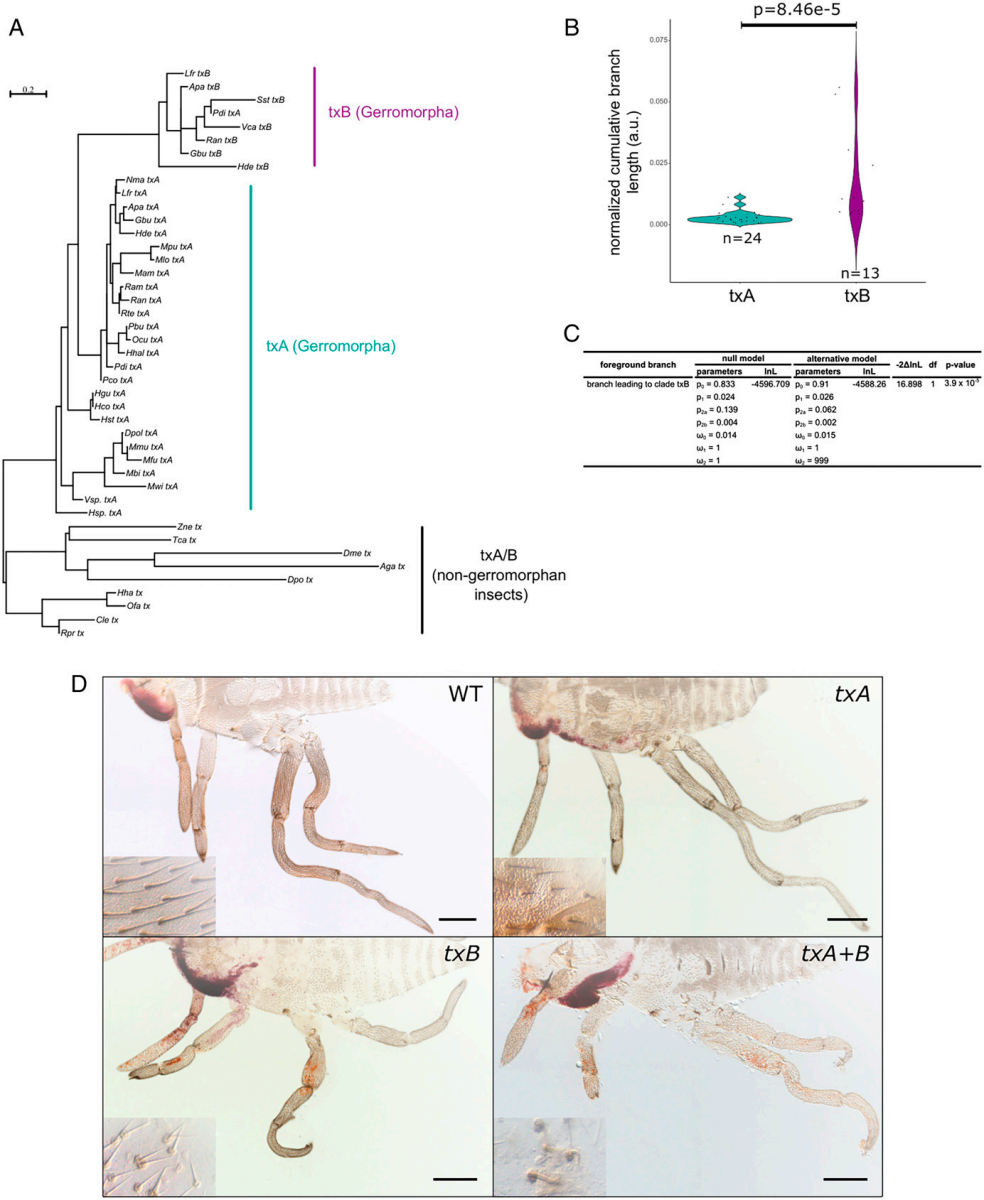
Duplication of the gene *tx* in the Gerromorpha. (*A*) Phylogram of *tx* homologs obtained through maximum-likelihood analysis using the LG+Γ+I model. Scale bar indicates the number of changes per site. (*B*) Comparative molecular evolution rates between txA and txB clades. (*C*) Statistics for the branch-site test of positive selection. These values were obtained by applying the branch-site test implemented in the package codeml of PAML. lnL: log likelihood score, −2ΔlnL: likelihood ratio test, and ω = d_N_/d_S_ (nonsynonymous/synonymous substitution rates). (*D*) Phenotypes of RNAi embryos in *G. buenoi*. The depletion of *txA*, *txB* or both simultaneously affected the development and organization of bristles on the body and the legs. The depletion of *txB* also caused a reduction of leg length. We obtained 34% (27 out of 79) of individuals with developmental defects for ds-*txA*, 27% (45 out of 164) for ds-*txB*, and 33% (23 out of 69) for the combined ds-*txA*/*txB*. WT: wild-type.

Among a list of more than 120 bristle genes we annotated in the genome of *G. buenoi* (20), *taxi* (*tx*), proved to be a rare example of duplication of a gene involved in bristle development. A phylogenetic reconstruction of the *tx* lineage across Heteroptera showed that the two copies *txA* and *txB* resulted from a gene duplication event that occurred at the node leading to the Gerromorpha, thus representing a Gerromorpha-restricted gene duplication (Fig. 2*A*). Analysis of coding sequence evolution revealed that *txB* underwent faster evolution compared to its paralog *txA* (Mann–Whitney *U* test, W = 89, *P* value = 8.46.10^−5^) (Fig. 2*B*). We also detected signatures of positive selection on the branch leading to the clade *txB* (likelihood ratio test [LRT], degree of freedom [df] = 1, *P* value = 3.9e-05) (Fig. 2*C*). These patterns of sequence evolution suggest that *txB* underwent functional divergence. RNAi against *txA*, *txB*, or both simultaneously caused the insects to develop lower-bristle density, disorganized bristle patterns, and generally shorter bristles on the body and the legs (Fig. 2*D*). We quantified bristle density in *txA* RNAi individuals, and we found that L1 legs (Kruskall–Wallis, df = 1, *P* value = 3.7e-4), L2 legs (Kruskall–Wallis, df = 1, *P* value = 2.8e-4), and L3 legs (Kruskall–Wallis, df = 1, *P* value = 6.6e-4) show an overall reduced bristle density (*SI Appendix*, Fig. S3*A*). We found the same trend in *txB* RNAi individuals with L1 legs (Kruskall–Wallis, df = 1, *P* value = 2.8e-4), L2 legs (Kruskall–Wallis, df = 1, *P* value = 1.6e-4), and L3 legs (Kruskall–Wallis, df = 1, *P* value = 1.7e-3) showing an overall reduced bristle density (*SI Appendix*, Fig. S3*B*). Moreover, the depletion of *txB* transcripts resulted in misoriented and/or abnormally adjacent bristles (Fig. 2 *D*, *Inset*). Surprisingly, RNAi against *txB*, but not *txA*, resulted in a severe reduction of leg length in addition to the bristle defects (Fig. 2*D*). These phenotypes suggest defects both in bristle patterning and leg length. In addition to the transcriptional silencing approach, we investigated the expression of *txA* and *txB* transcripts by in situ hybridization. Even though both genes are expressed ubiquitously, *txA* is expressed in the body and preferentially in the proximal region of the legs, whereas *txB* is visible in the whole legs and is generally stronger (*SI Appendix*, Fig. S4). These expression data reinforce the idea that *txA* and *txB* functionally diverged after duplication in the Gerromorpha.

**Fig. 3.**
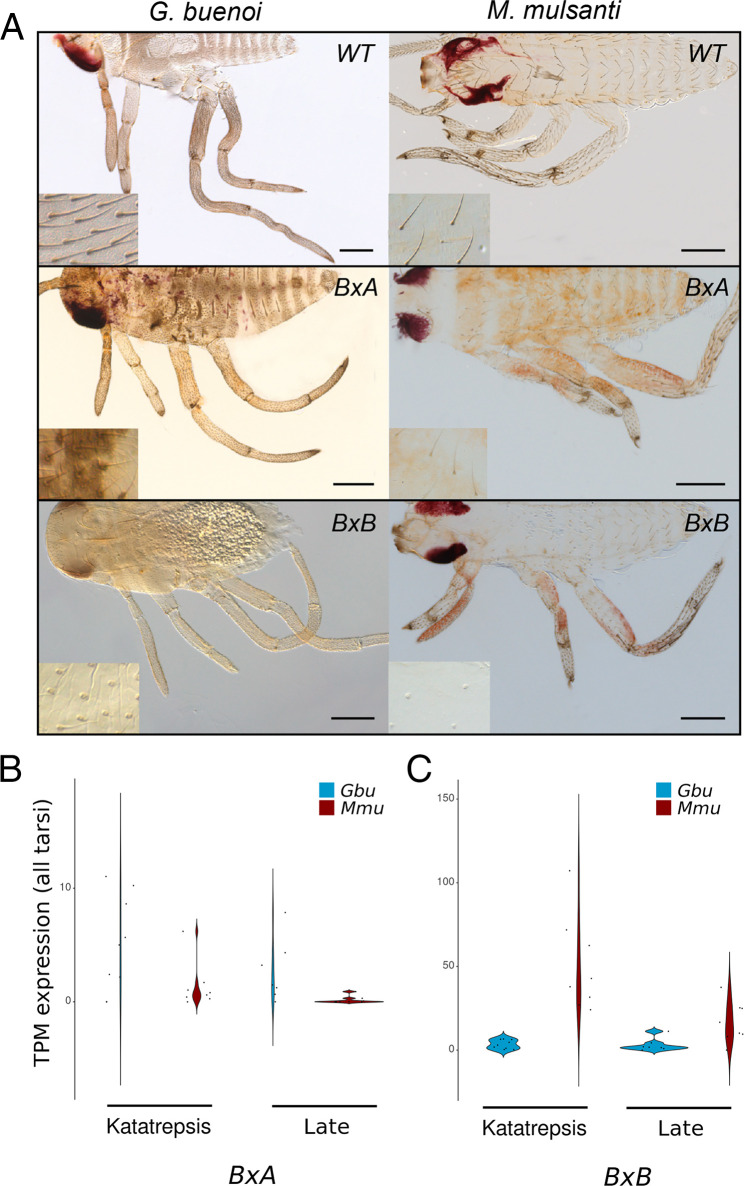
Duplication of the gene *Bx* in the Gerromorpha. (*A*) Phenotypes of RNAi embryos in *G. buenoi* and *M. mulsanti*. The depletion of *BxB* transcripts affected the development and the density of bristles in both species. The depletion of *BxA* only led to bristle phenotypes in *G. buenoi*. In *G. buenoi*, we obtained 32% (37 out of 117) of individuals with developmental defects for ds-*BxA* and 20% (20 out of 98) for ds-*BxB*. In *M. mulsanti*, we obtained 26% (20 out of 77) of individuals with developmental defects for ds-*BxA* and 23% (22 out of 96) for ds-*BxB*. Differences in the relative expression levels of the duplicates *BxA* (*B*) and *BxB* (*C*) in tarsi during embryogenesis in *G. buenoi* and *M. mulsanti*. The low expression of *BxA* in *M. mulsanti* might explain the absence of phenotype in RNAi embryos. WT: wild-type.

*Beadex* (*Bx*) is also present in two copies across the Gerromorpha, resulting from a duplication event that occurred before the divergence of the Hemiptera (*SI Appendix*, Fig. S5*A*). In *G. buenoi*, RNAi against *BxA* resulted in areas with lower density of bristles in the body and in the legs (Fig. 3*A*). The location of these low-bristle density areas varies among RNAi-treated embryos, suggesting that the variability we observe is a consequence of the RNAi technique, as reported in previous studies in the Gerromorpha (8, 9, 13). We quantified bristle density in *BxA* RNAi individuals, and we found that L1 legs (Kruskall–Wallis, df = 1, *P* value = 3.6e-4), L2 legs (Kruskall–Wallis, df = 1, *P* value = 3.7e-4), and L3 legs (Kruskall–Wallis, df = 1, *P* value = 3.7e-4) show an overall reduced bristle density (*SI Appendix*, Fig. S3*C*). Moreover, *BxA* RNAi embryos have shorter legs and do not hatch probably because of improper development of the nervous system (13). Embryos treated with *BxB* RNAi also showed bristle phenotypes, in which some bristles fail to extend the shafts in regions covering both the body and the legs of the embryos, even though the socket cells were always present (Fig. 3 *A*, *Inset*). We quantified bristle density in *BxB* RNAi individuals, and we found that L1 legs (Kruskall–Wallis, df = 1, *P* value = 1.1e-3), L2 legs (Kruskall–Wallis, df = 1, *P* value = 1.1e-3), and L3 legs (Kruskall–Wallis, df = 1, *P* value = 1.1e-3) show an overall reduced bristle density (*SI Appendix*, Fig. S3*D*). The cuticle also showed various defects, such as weaker pigmentation and higher fragility. The phenotypes obtained for the knockdown of *BxA* and *BxB* genes are reminiscent of the defects resulting from *ASH* depletion in the Gerromorpha (13). These results suggest that the function of *Bx* genes in activating the transcription of *ac*/*sc* genes (22) is conserved between Diptera and Gerromorpha. As observed for the gene *tx*, only one of the two copies (*BxA*) is implicated in regulating leg length in addition to its role in bristle development.

Next, we asked whether the two *Bx* paralogs perform a similar function in *M. mulsanti* that has a lower-leg bristle density. Whereas the depletion of *BxB* resulted in empty socket cells due to nonelongated bristles in *M. mulsanti* embryos (Fig. 3*A*), we failed to observe any phenotypes for double-stranded (ds)-*BxA* RNAi in this species. When we assessed the expression profile of *Bx* paralogs (Fig. 3 *B* and *C*) in our comparative transcriptomics datasets, we discovered that *BxA* is not significantly expressed during embryogenesis in *M. mulsanti* (expression < 2 transcripts per million [TPM]) (23), which is consistent with the absence of RNAi effect. These findings suggest that *bxA* and *bxB* emerged prior to the split of the Gerromorpha and that *BxA* acquired its function only later in the family Gerridae. Our analysis suggests that the difference between high- and low-bristle density observed in *G. buenoi* and *M. mulsanti* are attributable, at least partially, to differences in the expression of the gene *BxA*.

### Identifying Genes Involved in Bristle Density Using Comparative Transcriptomics.

To gain more insight into the genetic components regulating bristle density, we compared the transcriptomes of the developing tarsi during midembryogenesis and late embryogenesis (*SI Appendix*, Fig. S6*A*). This analysis is based on the hypothesis that the difference in bristle density between *G. buenoi* and *M. mulsanti* is the result of differences in the regulation of bristle patterning between these two species. We trimmed those lists by first removing lowly expressed transcripts (mean expression < 1 TPM) and then removing transcripts that are not differentially expressed between mid- and late embryogenesis in at least two legs. This analysis identified 7,578 and 7,166 transcripts that are differentially expressed between *G. buenoi* and *M. mulsanti* during midembryogenesis and late embryogenesis, respectively. BLAST searches led to the identification of 1,234 homologous sequences between *G. buenoi* and *M. mulsanti* (*SI Appendix*, Fig. S6 *B* and *C*). Among these 1,234 genes, we performed RNAi experiments for the genes showing higher fold-change and still expressed during postembryogenic stages at least in *G. buenoi*. The rationale is that bristle density significantly increases at stages N4 and N5 in *G. buenoi* contrary to *M. mulsanti* (Fig. 1*B*). Our RNAi-functional screening led to the identification of six genes that play a role in the development of bristles in *G. buenoi* and show an expression profile which is different between *G. buenoi* and *M. mulsanti* (*SI Appendix*, Fig. S6*D*). Below, we describe the role of a sample of these genes in water striders’ bristle development. Interestingly, these genes have not been previously associated with the development of bristles in the model species *Drosophila melanogaster*.

### Simiate: An Actin-Binding Protein.

**Fig. 4.**
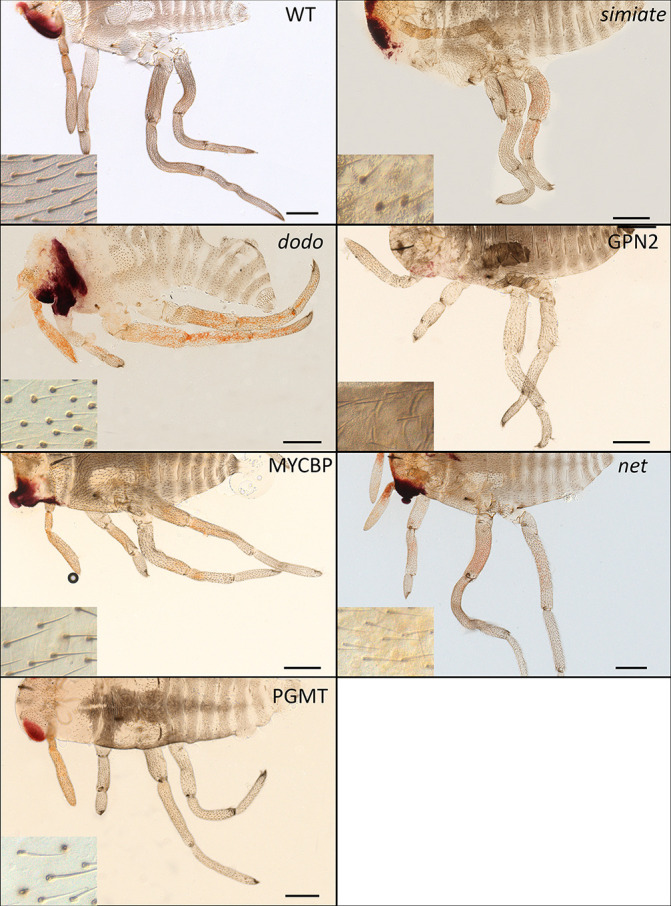
Functional characterization of relevant, differentially expressed genes in *G. buenoi*. *Simiate* RNAi knockdown resulted in disorganized and size variable bristles as well as shorter legs. *Dod* RNAi knockdown resulted in absent or nonelongated bristles (empty hair sockets) as well as shorter legs. *GPN2* RNAi knockdown resulted in absent or severely affected bristles as well as shorter legs. *PGMT* and *MYCBP* RNAi knockdown resulted in the reduced density of bristles as well as shorter legs. *Net* RNAi knockdown resulted in defective bristle elongation (empty hair sockets). The inset shows a magnified dorsal view of the abdomen. WT: wild-type. (Scale Bar, 200 μm.)

Among the differentially expressed genes, we identified the ortholog of the gene *Simiate* (*SI Appendix*, Fig. S5*B*). *Simiate* encodes an actin-binding protein that coordinates the arborization of neurons by acting on the actin cytoskeleton of filopodia (24). *Simiate* RNAi knockdown in *G. buenoi* resulted in embryos with bristles that are disorganized and variable in size, as well as shorter legs (Fig. 4 and *SI Appendix*, Fig. S7). Because the inner structure of bristle is made of actin filaments (25), *Simiate* might also act on actin polymerization and bristle orientation in the Gerromorpha.

### Dodo: A MAP Kinase Signal Responder Protein.

We also identified the ortholog of the gene *dodo* (*dod*) (*SI Appendix*, Fig. S5*C*). RNAi against *G. buenoi dod* resulted in embryos that are much shorter than wild-type embryos, without any clear anterior–posterior patterning defects. Milder phenotypes consist of body regions where only the sockets are visible, but the bristles are not elongated; regions where bristle density is lower; and/or patches devoid of bristles altogether (Fig. 4 and *SI Appendix*, Fig. S7). We quantified bristle density, and we found that L1 legs (Kruskall–Wallis, df = 1, *P* value = 1.7e-4), L2 legs (Kruskall–Wallis, df = 1, *P* value = 4.7e-2), and L3 legs (Kruskall–Wallis, df = 1, *P* value = 1.8e-2) show an overall reduced bristle density (*SI Appendix*, Fig. S3*E*). Interestingly, the legs of these embryos are shorter than control embryos (Fig. 4). These results demonstrate a role of *dod* both in bristle development and leg growth. We were also able to investigate the function of *dod* in *M. mulsanti*. *Dod* RNAi knockdown resulted in a moderate reduction in cuticle melanization, without apparent change in leg length or bristle density (*SI Appendix*, Fig. S8). These results suggest that the role of Dodo in controlling leg length and bristle density might be specific to *G. buenoi*.

### GPN-loop GTPase 2.

Our comparative, transcriptomic approach led to the identification of a GPN-loop GTPase, identified as GPN-loop GTPase 2 in our phylogenetic analysis (*SI Appendix*, Fig. S5*D*). In *G. buenoi*, *GPN2* depletion resulted in severe bristle defects on the body and the legs of the embryos. Some patchy regions of the embryos are devoid of bristles (Fig. 4 and *SI Appendix*, Fig. S7), a phenotype reminiscent of *Bx* (Fig. 3*A*) and *ASH* RNAi (13). We quantified bristle density, and we found that L1 legs (Kruskall–Wallis, df = 1, *P* value = 2.5e-4), L2 legs (Kruskall–Wallis, df = 1, *P* value = 3.7e-4), and L3 legs (Kruskall–Wallis, df = 1, *P* value = 3.9e-3) show an overall reduced bristle density (*SI Appendix*, Fig. S3*F*). Again, GPN2 RNAi also resulted in severe shortening of the legs (Fig. 4). These results suggest that the enzyme GPN2 plays a role early during the process of bristle patterning and leg growth in *G. buenoi* embryos. The molecular function of GPN-loop GTPases is still elusive, but the yeast enzyme Npa3 (a homolog of human GPN1) could function as an assembly chaperone for RNA polymerase II (26).

### Protein–Glutamate O-Methyltransferase.

We identified the gene that encodes the protein-glutamate O-methyltransferase (PGMT) as playing a role in bristle development in the Gerromorpha (*SI Appendix*, Fig. S5*E*). RNAi embryos do not hatch and exhibit a lower density of bristles along the body and legs in *G. buenoi* (Fig. 4 and *SI Appendix*, Fig. S7). We quantified bristle density, and we found that L1 legs (Kruskall–Wallis, df = 1, *P* value = 1.5e-4), L2 legs (Kruskall–Wallis, df = 1, *P* value = 1.6e-4), and L3 legs (Kruskall–Wallis, df = 1, *P* value = 1.6e-4) show an overall reduced bristle density (*SI Appendix*, Fig. S3*G*). We were also able to investigate the function of *PGMT* in *M. mulsanti*. *PGMT* RNAi knockdown also resulted in a strong reduction in cuticle melanization, without apparent change in leg length or bristle density (*SI Appendix*, Fig. S8). These results suggest that the role of PGMT in controlling leg length and bristle density might be specific to *G. buenoi*. Protein methyltransferases regulate important biological functions in eukaryotic cells through the posttranslational modification of a wide array of targets (27). These enzymes catalyze transfer of methyl groups from the cofactor *s*-adenosyl methionine to amine. Their biological role has been mainly investigated in prokaryotes. In eukaryotes, the human homolog Armt1 targets proliferating cell nuclear antigen (PCNA) in the context of DNA damage response (28).

### MYCBP: A c-Myc–Binding Protein.

We identified the ortholog of the gene *MYCBP* among the differentially expressed genes (*SI Appendix*, Fig. S5*F*). *MYCBP* RNAi embryos do not hatch and have a lower density of bristles on the antennae, the legs, and the head (Fig. 4 and *SI Appendix*, Fig. S7). We also noticed a shorter-leg phenotype among the rare mild RNAi embryos we obtained, most of the RNAi phenotypes being strong. c-Myc is a basic helix–loop–helix leucine zipper (bHLH-LZ) transcription factor that plays a key function in cell proliferation, differentiation, and apoptosis (reviewed in refs. 29 and 30).

### Net: A bHLH Transcription Factor.

Finally, we showed a role of the bHLH transcription factor Net in bristle elongation. Because *bHLH* genes encode a large superfamily in insects (31, 32), we reconstructed the phylogeny of this family to confirm the orthology relationships between our *G. buenoi* sequence and the gene lineage *net* (*SI Appendix*, Fig. S5*G*). In *G. buenoi*, depletion of *net* transcripts led to empty hair sockets on the legs and antennae of embryos (Fig. 4 and *SI Appendix*, Fig. S7). Previous studies revealed a role of Net during wing vein formation in *Drosophila*, in which Net represses the transcription of Rhomboid and antagonizes EGFR activity (33). However, our study reports the role of the bHLH factor Net in bristle development.

### Regulation of Rate and Orientation of Cell Division as a Common Mechanism for Leg Growth and Bristle Density.

**Fig. 5.**
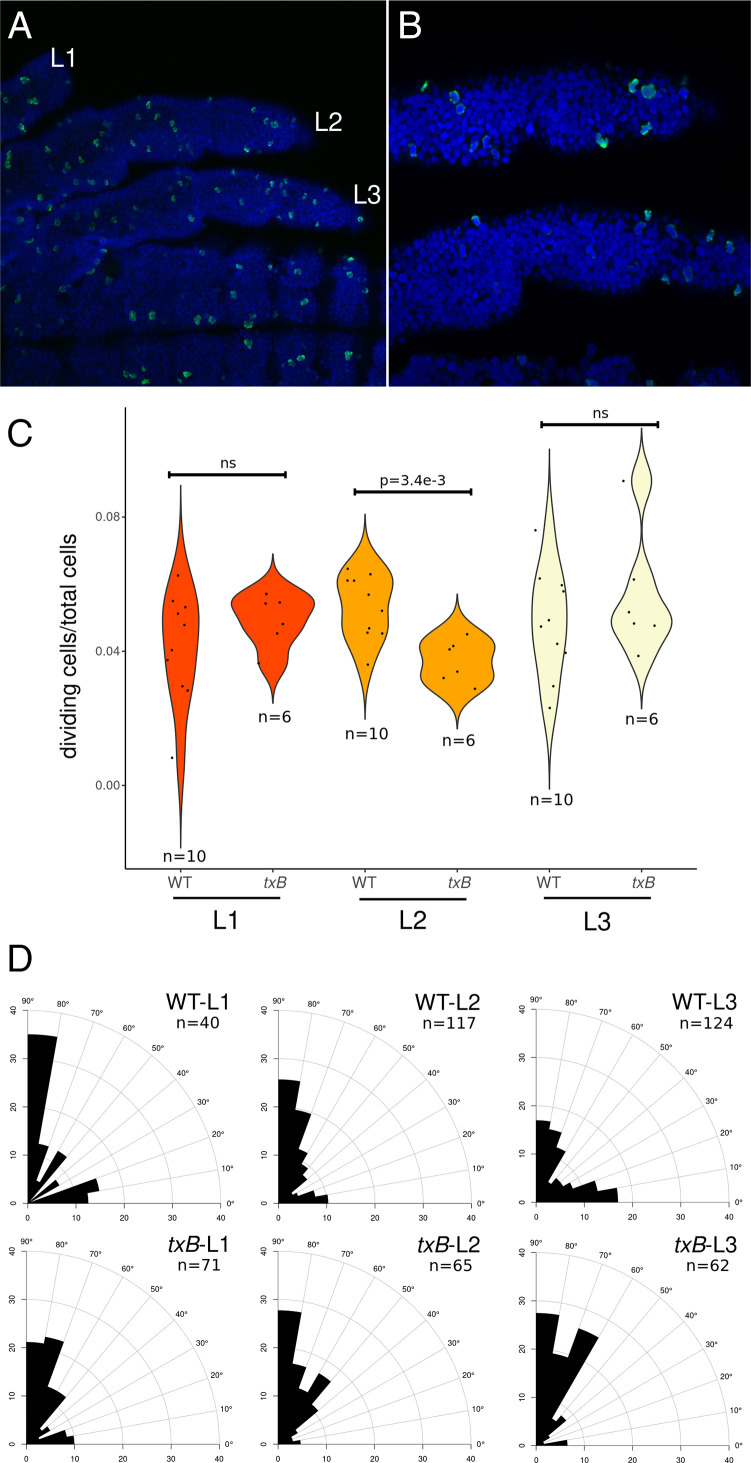
Division rate and orientation of cell division in *txB* RNAi individuals. (*A*) Wild-type (WT) embryo labeled with DAPI in blue (nuclei) and anti–Phospho-histone H3 in green (mitotic cells) observed with a 20× objective in confocal microscopy. (*B*) Same embryo observed with a 40× objective showing legs L2 and L3. (*C*) Quantification of the number of dividing cells over the total number of cells in the three different legs of WT and *txB* RNAi embryos. (*D*) Quantification of the angle of cell division in fixed embryos relative to the PD axis. The letter *n* indicates the number of cell divisions. ns: not significant.

RNAi knockdown of most genes analyzed resulted in both a decrease in leg length and bristle density. Given that cell division underlies leg growth in the Gerromorpha (*SI Appendix*, Fig. S2), we compared the rate of cell division in the legs between wild-type and *txB* RNAi individuals. In *G. buenoi*, the *txB* RNAi embryos have a significantly reduced bristle density on the three legs as well as shorter legs L2. We collected young embryos injected with ds-*txB* and labeled nuclei (DAPI) and mitotic cells (anti–phospho-histone H3) (Fig. 5 *A* and *B*). We quantified the ratio of dividing cells (PH3-positive cells), and we found a statistically significant decrease in the rate of cell division in the L2 legs of *txB* RNAi individuals (Kruskal–Wallis, df = 1, *P* value = 3.4e-3), but not in the L1 and L3 legs of the same individuals (Fig. 5*C*). The same trend was observed for leg length in ds-*txB* individuals, in which only L2 legs are shorter (*SI Appendix*, Fig. S3*B*). While quantifying division rate, we noticed possible bias in the orientation of cell division. This prompted us to quantify the orientation of cell division in relation with the proximodistal (PD) axis of the leg. We found that a reduced number of cells divide parallel to the PD axis, which could contribute to the overall reduction in growth of L2 legs in *txB* RNAi individuals (Fig. 5*D*). On the contrary, more cells divide perpendicular to the PD axis, which might explain slightly wider legs observed in *txB* RNAi individuals. In *Drosophila*, tx acts as a potent activator of βPS integrin expression (34), and it has been shown that integrin signaling regulates mitotic spindle orientation in epithelia (35, 36). Our data suggest that txB might have a similar cellular function in the *Gerris* legs. Further investigation would be required to test whether the other genes regulate leg length and bristle density through the same cellular mechanisms.

These results obtained through the RNAi-functional screen report the identification of six genes that are involved in the patterning, the elongation, as well as the spatial organization of bristles, in addition to a role in leg growth during *G. buenoi* embryogenesis. These genes are differentially expressed between early developmental stages in *G. buenoi* and *M. mulsanti*, suggesting that the regulation of their expression might be relevant at the macroevolutionary scale. Another drastic difference is that the expression of these six genes continues throughout postembryonic stages in *G. buenoi* but not in *M. mulsanti* (*SI Appendix*, Fig. S6*D*).

### The Patterning of Bristle Genes Is Still Active during Postembryonic Development.

**Fig. 6.**
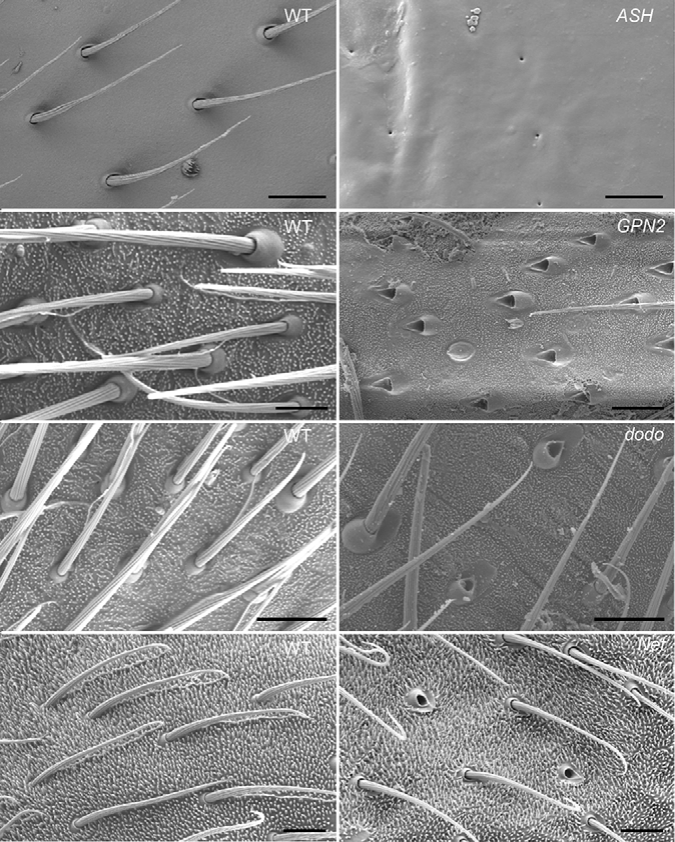
Phenotypes of RNAi-treated nymphs in *G. buenoi*. Besides impairments in molting and locomotion, the nymphs injected with ds-*ASH*, ds-*GPN2*, ds-*dod*, and ds-*net* exhibited bristle defects. These results show that the genes *ASH*, *GPN2*, *dod*, and *net* are still required to maintain the patterning and elongation of bristles during postembryonic development. WT: wild-type.

Because the dynamics of bristle density changes across postembryonic stages, we also depleted the candidate genes in the nymphs. First, we injected individuals with ds-*ASH* that produces defects in bristle patterning in several species of Gerromorpha when maternally depleted (13). We obtained individuals that died during the process of molting and exhibited bristle defects on the abdomen (Fig. 6). Besides this phenotype, impairments in locomotion behavior were observed in a few survivors that performed fast circular movements on the water surface. RNAi treatment indicated that the three genes, *GPN2*, *dod*, and *net*, maintain their role in bristle development during postembryonic stages. RNAi knockdown against all these genes resulted in bristle development defects consisting of missing leg bristles, despite the presence of the hair sockets or nonelongated bristles (Fig. 6).

These results indicate how a set of genes involved in patterning and cell division are redeployed to make new bristles during nymphal stages in *G. buenoi*. It also confirms our previous data on the dynamics of bristle density throughout development that already suggested a role for repatterning and cell division in increasing bristle density in *G. buenoi* (Fig. 1). Besides having a higher leg and body bristle density, water striders have significantly longer legs than their terrestrial relatives (5). In addition, L1 legs are shorter than L2 legs, which in turn are longer than L3 legs (8, 37). It is therefore possible that changes in the control of cell division might have enabled the evolution of both leg length and leg bristle density, both of which are important for exploiting surface tension and generation fast movement on the water–air interface.

### Most Genes that Play a Role in Bristle Density Are Also Required for Regulating Leg Length.

**Fig. 7.**
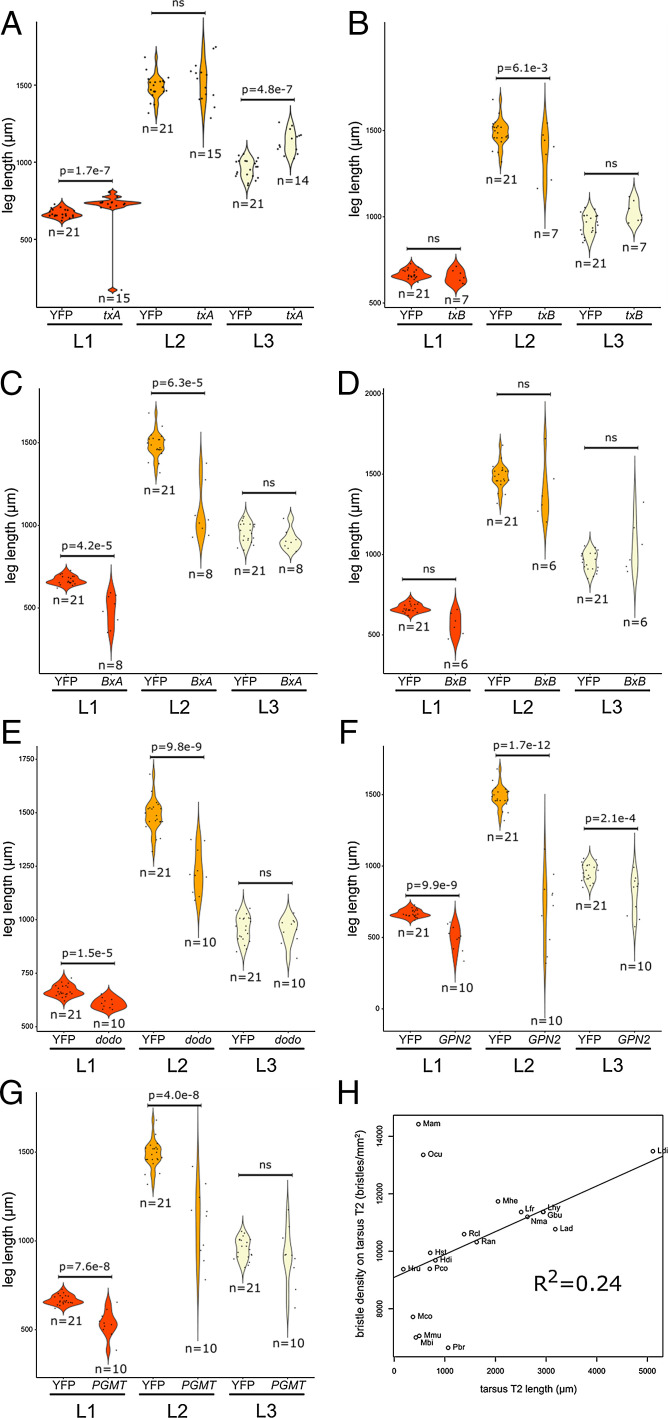
The same genes affect both bristle development and leg length. In *G. buenoi*, embryos depleted in *txA* (*A*), *txB* (*B*), *BxA* (*C*), *BxB* (*D*), *dod* (*E*), *GPN2* (*F*), and *PGMT* (*G*) transcripts show a dual phenotype: lower-leg bristle densities and shorter legs. (*H*) A positive correlation stands between bristle density in tarsus L2 and tarsus L2 length.

Because the knockdown of several genes we identified for their role in bristle development also resulted in shorter legs, we compared leg length in maternally injected RNAi versus control embryos in *G. buenoi*. The ds-*txA* embryos have significantly longer L1 (analysis of covariance [ANCOVA], *n* = 15, *P* value = 1.7e-07) and L3 legs (ANCOVA, *n* = 14, *P* value = 4.8e-07) (Fig. 7*A*). The ds-*txB* embryos have significantly shorter L2 legs (ANCOVA, *n* = 7, *P* value = 6.1e-03) (Fig. 7*B*). The ds-*BxA* embryos have significantly shorter L1 (ANCOVA, *n* = 8, *P* value = 4.2e-05) and L2 legs (ANCOVA, *n* = 8, *P* value = 6.3e-05) (Fig. 7*C*). The ds-*BxB* embryos followed the same trend with shorter L1 and L2 legs (Fig. 7*D*), but the difference was found statistically nonsignificant likely because of a limited number of samples (*n* = 6). The ds-*dod* embryos have significantly shorter L1 (ANCOVA, *n* = 10, *P* value = 1.5e-05) and L2 legs (ANCOVA, *n* = 10, *P* value = 9.8e-09) (Fig. 7*E*). The ds-*GPN2* embryos have significantly shorter L1 legs (ANCOVA, *n* = 10, *P* value = 9.9e-09), L2 legs (ANCOVA, *n* = 10, *P* value = 1.7e-12), and L3 legs (ANCOVA, *n* = 10, *P* value = 2.1e-04) (Fig. 7*F*). Lastly, the ds-*PGMT* embryos showed a similar trend with significantly shorter L1 legs (ANCOVA, *n* = 10, *P* value = 7.6e-08) and L2 legs (ANCOVA, *n* = 10, *P* value = 4e-08) (Fig. 7*G*). The lack of statistical significance in the decrease of leg length we observed in a handful of cases could be due to the incorporation of mild phenotypes in our analysis and/or small sample size. Another explanation is biological and might reside in the difference in the tissue landscape between the three legs. Cell division in L2 legs is more intense earlier during development, the time when RNAi effect is likely greatest than the two other legs, which could explain why these legs are often more affected in our experiments.

We also identified the shorter-leg phenotype after depletion of *MYCBP* and *Simiate*, but the RNAi was too severe and did not provide enough mildly affected embryos to perform robust statistical analyses. The implication of MYCBP in leg elongation is likely to occur in the light of its role in cell proliferation, differentiation, and apoptosis (reviewed in refs. 29 and 30). Whereas the function of Simiate as an actin-binding protein (24) explains the bristle defects in the phenocopies, it does not explain the shorter-leg phenotype. Besides its cytoplasmic role, Simiate can function as a transcription regulator and/or splicing enhancer in the mouse brain (38). This nuclear function of Simiate might underlie its direct or indirect implication in leg growth in the Gerromorpha.

These findings raise the hypothesis that increased bristle density evolved in concert with increased leg length, both of which are characteristic to the Gerromorpha (5). To further test this hypothesis, we reanalyzed our dataset of bristle density measurements in Gerromorpha L2 tarsi (Fig. 1) by adding the mean length of the tarsus L2 and the entire leg L2. We found that leg bristle density increases linearly with tarsus length (Pearson *R*^2^ = 0.24) (Fig. 7*H*). We also performed the same analysis of correlation for the control and knockdown individuals in *G. buenoi*. Our data showed that tarsus L2 bristle density positively correlates with tarsus L2 length (Pearson *R*^2^ = 0.41) (*SI Appendix*, Fig. S9). The strongest correlation found across knockdown individuals is likely due to the fact that the individuals are from the same species *G. buenoi*, which reduces the amount of cumulated lineage-specific divergence. These independent analyses reinforce the idea that the evolution of leg length and leg bristle density are correlated.

## Discussion

The ability of water striders to exploit surface tension and to adapt their leg morphologies to the hydrodynamics of the fluid substrate have been critical to their transition to life on the water surface. Andersen described this transition as a stepwise process including both the evolution of hydrophobic bristles and the diversification of locomotion morphology and behavior (1, 4). Our findings, however, suggest that these two traits are genetically correlated. In a previous study, we showed that diversity in locomotion behavior and morphology is associated with ecological niche expansion in the Gerromorpha (5). The Gerromorpha, generally, have longer legs than their terrestrial relatives, allowing them to achieve increased speed, and species that live in open-water habitats have longer legs than species occupying water margins (1, 5). The same species have also larger bodies (5) and higher leg bristle density (*SI Appendix*, Fig. S1) that could enhance their ability to maintain their body weight in open-water habitats.

The correlation between leg length and bristle density in water striders raises the question of whether common genetic and developmental mechanisms underlie these two traits. We suggest that this common mechanism consists of changes in cell division. First, we have shown that gene duplication plays a role in linking these two morphological traits. The Gerromorpha have two copies of the gene *Bx*, which is involved in bristle patterning in *D. melanogaster*. In line with this conclusion, the GATA transcription factor *pannier* (*pnr*) activates proneural expression during the development of dorsoventral bristles through binding to the DC enhancer of *ac*/*sc* (39), and Bx functions as a *pnr* coactivator to promote the expression of *ac*/*sc* genes (22). Moreover, the Gerromorpha have two copies of the gene *tx*, whose loss of function leads to ectopic bristles or hair cell duplication (34, 40). The same logic applies for both *Bx* and *tx* duplication, in that the two copies contribute to bristle patterning but only one copy regulates leg length. These findings suggest a link between leg bristle patterning and leg length. Interestingly, gene duplication was previously shown to be important for the evolutionary history of the Gerromorpha. The two taxon-restricted genes *geisha* and *mother-of-geisha*, which resulted from gene duplication, control the propelling fan necessary for the genus *Rhagovelia* to occupy fast-flowing streams (41). Altogether, these data suggest that specific duplications of genes important for bristle development may be associated with the adaptation of the Gerromorpha to life on the water–air interface.

Second, we identified genes that are important for bristle development in the Gerromorpha and that were not associated to bristle development in the model species *D. melanogaster*. We found that most of these genes are also required for the proper growth of the legs in *G. buenoi*. This finding reinforces the idea that leg bristle development and leg growth are genetically correlated in the Gerromorpha. Because the newly identified genes are involved in cell division, we propose that the regulation of cell division is a common mechanism underlying the evolution of the two adaptive traits in the Gerromorpha, namely, increased bristle density and leg length. For example, the cis-trans prolyl isomerase Dodo specifically targets the proline residue carboxyl-terminal to the phosphorylated threonine or serine residue and plays a role in kinase-mediated signal transduction pathways (MAPK) (42). Moreover, the orthologs in mammals (Pin1) and in yeast (Ess1/Ptf1) have been implicated in cell cycle control (43–46). Concerning the link between PGMT and leg growth, the human homolog Armt1 targets PCNA in the context of DNA damage response (28). It is possible that PCNA represents the link between PGMT and cell division in the Gerromorpha. Lastly, the link between MYCBP and cell division has been characterized in detail in various organisms. The bHLH motif allows c-Myc proteins to bind DNA, while the LZ TF–binding motif allows heterodimerization with another bHLH-LZ protein Max (47). c-Myc–Max dimers positively or negatively regulate a plethora of target genes (30), through the binding of the canonical E-boxes (48), variants thereof (49), or indirect and/or assisted DNA binding (50, 51).

The pleiotropic effects of a single gene or a genomic region are known to be important for morphological evolution and adaptation. In sticklebacks, the evolution of both skeletal development and sensory system phenotypes are associated with the gene *Ectodysplasin* (52). In *Drosophila*, the number of bristles in two copulatory organs, genitalia and sex combs, evolved in a correlated manner (53). Gould and Lewontin posited that some traits arose as by-products (“spandrels”) of developmental constraints on a crucial trait, without necessarily being a direct target of selection. As a result, evolutionary change is biased toward the phenotypic direction along which both traits change simultaneously. In water striders, the absence of species that have long legs with sparse bristles is compatible with a “spandrel” explanation. In the case of the Gerromorpha, it remains difficult to know whether the making of long legs involves denser bristles or whether the making of dense bristles involves longer legs. During development, the patterning of bristles is constrained by lateral inhibition (i.e., the capacity of a bristled cell to inhibit the specification of bristles in neighbor cells) that is mediated by the Notch signaling pathway. Hence, increasing the number of cells becomes a way to evolve denser bristles. The fact that having longer legs, or denser bristles, may have originally been an indirect target of selection does not exclude that it has later imparted a selective advantage when the habitat changed. The evolution of bristle patterns was necessary for exploiting surface tension and leg length necessary for generating high speed on water, thus promoting the diversification of this group of insects on the water–air interface.

## Materials and Methods

### Animal Collection and Rearing.

*G. buenoi* were collected at the vicinity of Toronto, Ontario, Canada. *M. mulsanti* were collected in a pond near Cayenne, French Guiana. Water striders were kept in aquaria at 25 °C with a 14-h light/10-h dark cycle and were fed on live crickets. Pieces of floating Styrofoam were regularly supplied to female water striders to lay eggs.

### Scanning Electron Microscopy.

Nymphs or adult bugs were sputter coated (Baltec MED-020, Leica Microsystems GmbH) with 10 nm copper. Samples were examined by using a Hitachi S800 SEM.

### Weight Measurement.

Insects were individually weighed with an A&D BM-20 analytical balance to a precision of 10^−6^ g when the weight was detectable. Otherwise, 10 nymphs (*G. buenoi* stage N1 and *M. mulsanti* stage N2) or 20 nymphs (*M. mulsanti* stage N1) were pooled to obtain an average weight of the corresponding developmental stage. A total of 10 individuals per condition were measured.

### Leg Measurements.

We compared the leg length between RNAi-injected embryos and ds-*YFP*–injected embryos (negative control). For each late embryo, we measured both the length of the chorionated embryo and the length of the three legs. Once the measure of the embryo length completed, embryos were dechorionated and mounted on slides in Hoyer’s medium. Legs were photographed and measured using a Zeiss AxioVision microscope (Zen software).

### Quantifying Bristle Density.

Images of embryonic legs were captured under bright-field illumination by using a Zeiss AxioObserver Z1 inverted microscope. Images of nymphal and adult legs were captured by using a Hitachi S800 scanning electron microscopy. Bristles were manually counted using the tool PointPicker implemented in Fiji (54).

### Quantifying Cell Density.

Tarsi were dissected in phosphate buffer saline (PBS) 1%, fixed in paraformaldehyde 4% for 20 min, and then treated in sucrose 30% for 30 min, and tissues were finally embedded in freezing medium NEG-50 (Thermo Fisher Scientific) following immersion in liquid nitrogen. Tissues were longitudinally cut using a cryostat Leica CM3050 S and stained with DAPI (Invitrogen D1306) overnight. Tissues were mounted in Vectashield (Vector Laboratories) and then observed by using a confocal microscope Zeiss LSM 780. Nuclei were manually counted using the tool PointPicker implemented in Fiji (54).

### Analysis of Orientation of Cell Division in Fixed Embryos.

Embryos were dechorionated in 1.3% bleach diluted in PBS 0.05% Tween 20 (PTW) and rinsed in PTW. Embryos were then fixed in 4% formaldehyde for 20 min, washed three times in PTW, and permeabilized in PBS 0.3% Triton-X-100 (PBT) for 10 min. Permeabilized embryos were blocked in 1% bovine serum albumin (BSA) in PBT for 1 h and incubated overnight with the primary antibody rabbit anti–phospho-histone H3 [Santa Cruz Biotechnology, p-Histone H3 (Ser-10)-R, 1:1,000]. Embryos were washed two times in PTW for 10 min before incubation for 2 h at room temperature with the secondary antibody goat anti-rabbit (Invitrogen, Alexa Fluor Plus 488, 1:500). Finally, embryos were washed two times in PBT, three more times in PTW, and incubated in glycerol 50% DAPI for 5 min. Embryos were then mounted in Vectashield and observed using a confocal microscope Zeiss LSM 780 with a 40× objective.

Only cells in anaphase or telophase were analyzed to ensure that the spindles had finished rotation before fixation. For each cell division, the angle α_PD_ between the local PD axis of the leg and the separating chromosomes was measured using the angle tool in Fiji. Angles were always measured as acute angles. To obtain the orientation of cell division in the relation to the PD axis, the angle α_PD_ was transformed as follows: α_division_ = 90° −α_PD_. Angular histograms were plotted using an in-house R script available at the Dryad digital depository database (https://doi.org/10.5061/dryad.fbg79cns8).

### Sequence Data Collection.

Phylogenetic markers were identified in available genomes or transcriptomes by tBLASTn using a set of selected *D. melanogaster* genes as a probe. Species names are indicated by the following prefixes Aga: *Anopheles gambiae*, Apa: *Aquarius paludum*, Api: *Acyrthosiphon pisum*, Bal: *Brachymetra albinervus*, Bge: *Blattella germanica*, Bta: *Bemisia tabaci*, Cle: *Cimex lectularius*, Dme: *D. melanogaster*, Dno: *Diuraphis noxia*, Dpo: *Dendroctonus ponderosae*, Dpol: *Darwinivelia polhemi*, Gbu: *G. buenoi*, Gmo: *Glossina morsitans*, Hco: *Hydrometra comata*, Hdi: *Husseyella diffidens*, Hgu: *Hydrometra guianana*, Hha: *Halyomorpha halys*, Hhal: *Husseyella halophila*, Hru: *Hebrus ruficeps*, Hst: *Hydrometra stagnorum*, Htu: *Husseyella turmalis*, Hvi: *Homalodisca vitripennis*, Lad: *Limnogonus aduncus*, Ldi: *L. dissortis*, Lfr: *Limnogonus franciscanus*, Lhu: *Linepithema humile*, Lhy: *Limnogonus hyalinus*, Mam: *Microvelia americana*, Mbi: *Mesovelia bila*, Mco: *Microvelia cortipes*, Mfu: *Mesovelia furcata*, Mhe: *Metrobates hesperius*, Mlo: *Microvelia longipes*, Mmu: *M. mulsanti*, Mpu: *Microvelia pulchella*, Mwi: *Mesoveloidea williamsi*, Nma: *Neogerris magnus*, Ocu: *Oiovelia cunucunumana*, Ofa: *Oncopeltus fasciatus*, Pbr: *Platyvelia brachialis*, Pbu: *Paravelia bullialata*, Pco: *Paravelia conata*, Pdi: *Paravelia dilatata*, Ram: *Rhagovelia amazonensis*, Ran: *Rhagovelia antilleana*, Rcl: *Rheumatobates clanis*, Rpr: *Rhodnius prolixus*, Rte: *Rhagovelia tenuipes*, Sst: *Stridulivelia strigosa*, Tca: *Tribolium castaneum*, and Zne: *Zootermopsis nevadensis*. The sequences generated for this analysis have been deposited in the European Molecular Biology Laboratory database with specific accession numbers (*SI Appendix*, Table S1).

### Phylogenetic Analysis.

Amino acid sequences were aligned with MUSCLE (55) and manually adjusted, and selected blocks were used for phylogenetic reconstruction. Maximum-likelihood searches were performed using RAxML (56) under the LG+Γ+I model. A total of 1,000 bootstrap replicates were conducted for support estimation. The different sequence alignments and tree files are downloadable from the Dryad digital depository database (https://doi.org/10.5061/dryad.fbg79cns8).

### Detection of Positive Selection.

Candidate genes were tested for signatures of positive selection based on the ratio ω = d_N_/d_S_ (nonsynonymous/synonymous substitution rates) using the program codeml of PAML version 4.8 (57). We compared the null model (ω fixed to 1) to the alternative branch-site model that allows some sites to have an ω greater than one in some branches. The two models were compared using an LRT. A *P* value <0.05 means that the model with positive selection better explains the data. The codon alignment, used as input in PAML, was generated using the software PAL2NAL (58).

### Statistical Analysis.

The density of tarsal bristles was quantified in 10 Heteroptera species, including representatives of terrestrial bugs (group 1), early-diverging lineages of Gerromorpha (group 2), and derived lineages of Gerromorpha (group 3). A total of ∼10 individuals per species were collected. We tested the group effect using a Kruskal–Wallis test, as the density did not follow a normal distribution.

The cumulative branch length (CBL) for the clades txA and txB was calculated by adding all branch lengths within a clade. Branch lengths were obtained as outputs of RAxML software (56). To consider differences in the number of sequences per clade, we calculated normalized CBL that is the value of CBL/number of sequences per clade. We compared the CBL means between the clades txA and txB using a Mann–Whitney *U* test, as the CBL did not follow a normal distribution.

We tested for difference in leg length between RNAi-treated and control embryos by applying an ANCOVA. Egg length was used as a covariate as a possible factor impacting leg length, and the mean value of each pair of leg segments was used as the dependent variable. Prior to the ANCOVA test, the normal distribution of the data were checked with a Shapiro test.

Statistical tests were performed using R statistics package version 3.5.0 (the R Project for Statistical Computing, http://www.r-project.org).

### Quantifying Gene Expression.

Total RNA was extracted from fourth- and fifth-instar nymph tarsi using the miRNeasy kit (Qiagen) suitable for small amount of tissue. Embryo tarsi were treated differently by undergoing a cell lysis step instead of RNA extraction. RNA sequencing libraries were prepared by using a low-input SMARTer kit (Clontech), and deep sequencing was performed by using HiSEq. 4000 technology (Illumina). An average of 80 million reads was generated per sample. The reads were trimmed and filtered to remove low-quality bases and mapped to a reference transcriptome assembly of *G. buenoi* or *M. mulsanti*. The reference transcriptome assemblies of *G. buenoi* and *M. mulsanti* were obtained with the software Trinity (59). The read count for each transcript was calculated using RSEM (60). A dedicated R script has been written to obtain the differentially expressed genes from RSEM read counts using the DESeq2 package (61). To compare gene expression between orthologs in *G. buenoi* and *M. mulsanti*, the differentially expressed genes in *M. mulsanti* were blasted against *G. buenoi* transcriptome. Both *M. mulsanti* and *G. buenoi* transcriptomes were blasted against the nonredundant protein database, and Gene Ontology (GO) terms were found with Blast2GO (62). The raw reads of *G. buenoi* and *M. mulsanti* tarsus transcriptomes have been deposited in National Center for Biotechnology Information with the following accession numbers: ERZ778085.

### Maternal and Nymphal RNAi.

*Gerris* and *Mesovelia* RNAi were performed as previously described (8). After injection, *Mesovelia* embryos were placed at 31 °C to allow them to develop faster and thus obtain stronger phenotypes (13). Forward and reverse primers, both containing the T7 RNA polymerase promoter used to generate double-stranded RNA, are listed in *SI Appendix*, Table S2. Counts of RNAi effect per gene can be found in *SI Appendix*, Table S3.

### In Situ Hybridization.

Dissected embryos were fixed in 4% paraformaldehyde:heptane (ratio 1:3) for 20 min at room temperature, washed several times in cold methanol, and then rehydrated through a methanol series to PTW. Embryos were prehybridized for an hour at 60 °C in hybridization buffer (for composition see ref. 8) prior to the addition of digoxigenin-labeled RNA probe overnight at 60 °C. Blocking step was performed in 1% BSA prior to incubation with anti-digoxigenin antibody coupled with alkaline phosphatase for 2 h at room temperature. Embryos were washed several times before revelation with nitro-blue tetrazolium chloride/5-bromo-4-chloro-3′-indolyphosphate in alkaline phosphatase buffer. Embryos were mounted on slides in Hoyer’s medium and photographed on a Zeiss Axio observer microscope.

## Supplementary Material

Supplementary File

## Data Availability

Sequence data have been deposited in NCBI (ERZ778085). All other study data are included in the article and/or *SI Appendix*.

## References

[1] N. M. Andersen, The Semiaquatic Bugs (Hemiptera: Gerromorpha) (Scandinavian Science Press Ltd., Klampenborg, Denmark, 1982), vol. 3.

[2] L. Cheng, “Marine insects and the sea-skater Halobates (Hemiptera: Gerridae)” in Encyclopedia of Entomology, J. L. Capinera, Ed. (Springer, Dordrecht, 2004).

[3] J. T. Polhemus, D. A. Polhemus, “Global diversity of true bugs (Heteroptera; Insecta) in freshwater” in Freshwater Animal Diversity Assessment, vol. 198, E. V. Balian, C. Lévêque, H. Segers, K. Martens, Eds. (Springer, Dordrecht, 2007), pp. 379–391.

[4] N. M. Andersen, A comparative study of locomotion on the water surface in semiaquatic bugs (insecta, hemiptera, gerromorpha). Videnskabelige Meddelelser Dansk Naturhistorisk Forening 139, 337–396 (1976).

[5] A. J. J. Crumière , Diversity in morphology and locomotory behavior is associated with niche expansion in the semi-aquatic bugs. Curr. Biol. 26, 3336–3342 (2016). 27939311 10.1016/j.cub.2016.09.061 PMC5196023

[6] X. Gao, L. Jiang, Biophysics: Water-repellent legs of water striders. Nature 432, 36 (2004). 15525973 10.1038/432036a

[7] D. L. Hu, J. W. M. Bush, The hydrodynamics of water-walking arthropods. J. Fluid Mech. 644, 5–33 (2010).

[8] A. Khila, E. Abouheif, L. Rowe, Evolution of a novel appendage ground plan in water striders is driven by changes in the Hox gene Ultrabithorax. PLoS Genet. 5, e1000583 (2009). 19649305 10.1371/journal.pgen.1000583 PMC2709915

[9] P. N. Refki, D. Armisén, A. J. Crumière, S. Viala, A. Khila, Emergence of tissue sensitivity to Hox protein levels underlies the evolution of an adaptive morphological trait. Dev. Biol. 392, 441–453 (2014). 24886828 10.1016/j.ydbio.2014.05.021 PMC4111901

[10] X.-Q. Feng, X. Gao, Z. Wu, L. Jiang, Q.-S. Zheng, Superior water repellency of water strider legs with hierarchical structures: Experiments and analysis. Langmuir 23, 4892–4896 (2007). 17385899 10.1021/la063039b

[11] Y. Xue, H. Yuan, W. Su, Y. Shi, H. Duan, Enhanced load-carrying capacity of hairy surfaces floating on water. Proc. Math. Phys. Eng. Sci. 470, 20130832 (2014). 24808757 10.1098/rspa.2013.0832 PMC3973396

[12] Q. Wang, X. Yao, H. Liu, D. Quéré, L. Jiang, Self-removal of condensed water on the legs of water striders. Proc. Natl. Acad. Sci. U.S.A. 112, 9247–9252 (2015). 26170300 10.1073/pnas.1506874112 PMC4522788

[13] C. Finet, A. Decaras, D. Armisén, A. Khila, The *achaete-scute* complex contains a single gene that controls bristle development in the semi-aquatic bugs. Proc. Biol. Sci. 285, 20182387 (2018). 30487316 10.1098/rspb.2018.2387 PMC6283939

[14] S. Romani, S. Campuzano, E. R. Macagno, J. Modolell, Expression of achaete and scute genes in *Drosophila* imaginal discs and their function in sensory organ development. Genes Dev. 3, 997–1007 (1989). 2777079 10.1101/gad.3.7.997

[15] P. Cubas, J. F. de Celis, S. Campuzano, J. Modolell, Proneural clusters of achaete-scute expression and the generation of sensory organs in the *Drosophila* imaginal wing disc. Genes Dev. 5, 996–1008 (1991). 2044965 10.1101/gad.5.6.996

[16] J. B. Skeath, S. B. Carroll, Regulation of achaete-scute gene expression and sensory organ pattern formation in the *Drosophila* wing. Genes Dev. 5, 984–995 (1991). 2044964 10.1101/gad.5.6.984

[17] S. R. Wheeler, M. L. Carrico, B. A. Wilson, S. J. Brown, J. B. Skeath, The expression and function of the achaete-scute genes in *Tribolium castaneum* reveals conservation and variation in neural pattern formation and cell fate specification. Development 130, 4373–4381 (2003). 12900453 10.1242/dev.00646

[18] L. Dufour, “Recherches anatomiques et physiologiques sur les Hémiptères, accompagnées de considérations relatives à l’histoire naturelle et à la classification de ces insectes” in Mémoires des savants étrangers (Imprimerie de Bachelier, Paris, 1833), pp. 68–74.

[19] N. M. Andersen, Fine structure of the body hair layers and morphology of the spiracles of semiaquatic bugs in relation to life on the water surface. Videnskabelige Meddelelser Dansk Naturhistorisk Forening 140, 7–37 (1977).

[20] D. Armisén , The genome of the water strider *Gerris buenoi* reveals expansions of gene repertoires associated with adaptations to life on the water. BMC Genomics 19, 832 (2018). 30463532 10.1186/s12864-018-5163-2 PMC6249893

[21] D. L. Hu, B. Chan, J. W. M. Bush, The hydrodynamics of water strider locomotion. Nature 424, 663–666 (2003). 12904790 10.1038/nature01793

[22] J. Asmar, I. Biryukova, P. Heitzler, *Drosophila* dLMO-PA isoform acts as an early activator of achaete/scute proneural expression. Dev. Biol. 316, 487–497 (2008). 18329012 10.1016/j.ydbio.2008.01.040

[23] R. Dean , Sperm competition shapes gene expression and sequence evolution in the ocellated wrasse. Mol. Ecol. 26, 505–518 (2017). 27862511 10.1111/mec.13919

[24] K. Derlig , Simiate is an actin binding protein involved in filopodia dynamics and arborization of neurons. Front. Cell. Neurosci. 8, 99 (2014). 24782708 10.3389/fncel.2014.00099 PMC3986562

[25] J. Overton, The fine structure of developing bristles in wild type and mutant *Drosophila melanogaster*. J. Morphol. 122, 367–379 (1967). 6050992 10.1002/jmor.1051220406

[26] J. Niesser, F. R. Wagner, D. Kostrewa, W. Mühlbacher, P. Cramer, Structure of GPN-loop GTPase Npa3 and implications for RNA polymerase II assembly. Mol. Cell. Biol. 36, 820–831 (2015). 26711263 10.1128/MCB.01009-15 PMC4760217

[27] M. A. Grillo, S. Colombatto, S-adenosylmethionine and protein methylation. Amino Acids 28, 357–362 (2005). 15838589 10.1007/s00726-005-0197-6

[28] J. J. P. Perry , Human C6orf211 encodes Armt1, a protein carboxyl methyltransferase that targets PCNA and is linked to the DNA damage response. Cell Rep. 10, 1288–1296 (2015). 25732820 10.1016/j.celrep.2015.01.054 PMC4350021

[29] S. Pelengaris, M. Khan, The many faces of c-MYC. Arch. Biochem. Biophys. 416, 129–136 (2003). 12893289 10.1016/s0003-9861(03)00294-7

[30] T. R. Kress, A. Sabò, B. Amati, MYC: Connecting selective transcriptional control to global RNA production. Nat. Rev. Cancer 15, 593–607 (2015). 26383138 10.1038/nrc3984

[31] A. W. Moore, S. Barbel, L. Y. Jan, Y. N. Jan, A genomewide survey of basic helix-loop-helix factors in *Drosophila*. Proc. Natl. Acad. Sci. U.S.A. 97, 10436–10441 (2000). 10973473 10.1073/pnas.170301897 PMC27042

[32] V. Ledent, O. Paquet, M. Vervoort, Phylogenetic analysis of the human basic helix-loop-helix proteins. Genome Biol. 3, RESEARCH0030 (2002). 12093377 10.1186/gb-2002-3-6-research0030 PMC116727

[33] D. Brentrup, H. Lerch, H. Jäckle, M. Noll, Regulation of *Drosophila* wing vein patterning: Net encodes a bHLH protein repressing rhomboid and is repressed by rhomboid-dependent Egfr signalling. Development 127, 4729–4741 (2000). 11023875 10.1242/dev.127.21.4729

[34] N. Egoz-Matia , Spatial regulation of cell adhesion in the *Drosophila* wing is mediated by Delilah, a potent activator of βPS integrin expression. Dev. Biol. 351, 99–109 (2011). 21215259 10.1016/j.ydbio.2010.12.039

[35] A. Fernández-Miñán, M. D. Martín-Bermudo, A. González-Reyes, Integrin signaling regulates spindle orientation in *Drosophila* to preserve the follicular-epithelium monolayer. Curr. Biol. 17, 683–688 (2007). 17363255 10.1016/j.cub.2007.02.052

[36] F. Toyoshima, E. Nishida, Integrin-mediated adhesion orients the spindle parallel to the substratum in an EB1- and myosin X-dependent manner. EMBO J. 26, 1487–1498 (2007). 17318179 10.1038/sj.emboj.7601599 PMC1829369

[37] A. Khila, E. Abouheif, L. Rowe, Comparative functional analyses of Ultrabithorax reveal multiple steps and paths to diversification of legs in the adaptive radiation of semi-aquatic insects. Evolution 68, 2159–2170 (2014). 24766229 10.1111/evo.12444

[38] K. Derlig, A. Gießl, J. H. Brandstätter, R. Enz, R. Dahlhaus, Identification and characterisation of Simiate, a novel protein linked to the fragile X syndrome. PLoS One 8, e83007 (2013). 24349419 10.1371/journal.pone.0083007 PMC3859600

[39] M. J. García-García, P. Ramain, P. Simpson, J. Modolell, Different contributions of pannier and wingless to the patterning of the dorsal mesothorax of *Drosophila*. Development 126, 3523–3532 (1999). 10409499 10.1242/dev.126.16.3523

[40] J. L. Mummery-Widmer , Genome-wide analysis of Notch signalling in *Drosophila* by transgenic RNAi. Nature 458, 987–992 (2009). 19363474 10.1038/nature07936 PMC2988197

[41] M. E. Santos, A. Le Bouquin, A. J. J. Crumière, A. Khila, Taxon-restricted genes at the origin of a novel trait allowing access to a new environment. Science 358, 386–390 (2017). 29051384 10.1126/science.aan2748

[42] T. Hsu, D. McRackan, T. S. Vincent, H. Gert de Couet, *Drosophila* Pin1 prolyl isomerase Dodo is a MAP kinase signal responder during oogenesis. Nat. Cell Biol. 3, 538–543 (2001). 11389437 10.1038/35078508

[43] K. P. Lu, S. D. Hanes, T. Hunter, A human peptidyl-prolyl isomerase essential for regulation of mitosis. Nature 380, 544–547 (1996). 8606777 10.1038/380544a0

[44] S. D. Hanes, P. R. Shank, K. A. Bostian, Sequence and mutational analysis of ESS1, a gene essential for growth in *Saccharomyces cerevisiae*. Yeast 5, 55–72 (1989). 2648698 10.1002/yea.320050108

[45] D. G. Crenshaw, J. Yang, A. R. Means, S. Kornbluth, The mitotic peptidyl-prolyl isomerase, Pin1, interacts with Cdc25 and Plx1. EMBO J. 17, 1315–1327 (1998). 9482729 10.1093/emboj/17.5.1315 PMC1170480

[46] M. Shen, P. T. Stukenberg, M. W. Kirschner, K. P. Lu, The essential mitotic peptidyl-prolyl isomerase Pin1 binds and regulates mitosis-specific phosphoproteins. Genes Dev. 12, 706–720 (1998). 9499405 10.1101/gad.12.5.706 PMC316589

[47] E. M. Blackwood, R. N. Eisenman, Max: A helix-loop-helix zipper protein that forms a sequence-specific DNA-binding complex with Myc. Science 251, 1211–1217 (1991). 2006410 10.1126/science.2006410

[48] A. Sabò, B. Amati, Genome recognition by MYC. Cold Spring Harb. Perspect. Med. 4, a014191 (2014). 24492846 10.1101/cshperspect.a014191 PMC3904094

[49] M. Eilers, R. N. Eisenman, Myc’s broad reach. Genes Dev. 22, 2755–2766 (2008). 18923074 10.1101/gad.1712408 PMC2751281

[50] S. Neph , An expansive human regulatory lexicon encoded in transcription factor footprints. Nature 489, 83–90 (2012). 22955618 10.1038/nature11212 PMC3736582

[51] A. Soufi , Pioneer transcription factors target partial DNA motifs on nucleosomes to initiate reprogramming. Cell 161, 555–568 (2015). 25892221 10.1016/j.cell.2015.03.017 PMC4409934

[52] M. G. Mills, A. K. Greenwood, C. L. Peichel, Pleiotropic effects of a single gene on skeletal development and sensory system patterning in sticklebacks. Evodevo 5, 5 (2014). 24499504 10.1186/2041-9139-5-5 PMC3976036

[53] O. Nagy , Correlated evolution of two copulatory organs via a single cis-regulatory nucleotide change. Curr. Biol. 28, 3450–3457.e13 (2018). 30344115 10.1016/j.cub.2018.08.047 PMC7385753

[54] J. Schindelin , Fiji: An open-source platform for biological-image analysis. Nat. Methods 9, 676–682 (2012). 22743772 10.1038/nmeth.2019 PMC3855844

[55] R. C. Edgar, MUSCLE: Multiple sequence alignment with high accuracy and high throughput. Nucleic Acids Res. 32, 1792–1797 (2004). 15034147 10.1093/nar/gkh340 PMC390337

[56] A. Stamatakis, RAxML version 8: A tool for phylogenetic analysis and post-analysis of large phylogenies. Bioinformatics 30, 1312–1313 (2014). 24451623 10.1093/bioinformatics/btu033 PMC3998144

[57] Z. Yang, PAML 4: Phylogenetic analysis by maximum likelihood. Mol. Biol. Evol. 24, 1586–1591 (2007). 17483113 10.1093/molbev/msm088

[58] M. Suyama, D. Torrents, P. Bork, PAL2NAL: Robust conversion of protein sequence alignments into the corresponding codon alignments. Nucleic Acids Res. 34, W609–W6012 (2006). 16845082 10.1093/nar/gkl315 PMC1538804

[59] M. G. Grabherr , Full-length transcriptome assembly from RNA-Seq data without a reference genome. Nat. Biotechnol. 29, 644–652 (2011). 21572440 10.1038/nbt.1883 PMC3571712

[60] B. Li, C. N. Dewey, RSEM: Accurate transcript quantification from RNA-Seq data with or without a reference genome. BMC Bioinformatics 12, 323 (2011). 21816040 10.1186/1471-2105-12-323 PMC3163565

[61] M. I. Love, W. Huber, S. Anders, Moderated estimation of fold change and dispersion for RNA-seq data with DESeq2. Genome Biol. 15, 550 (2014). 25516281 10.1186/s13059-014-0550-8 PMC4302049

[62] S. Götz , High-throughput functional annotation and data mining with the Blast2GO suite. Nucleic Acids Res. 36, 3420–3435 (2008). 18445632 10.1093/nar/gkn176 PMC2425479

