# Supplementary material for: Leg length and bristle density, both necessary for water surface locomotion, are genetically correlated in water striders
Source: Proc Natl Acad Sci U S A. 2022 Feb 22;119(9):e2119210119. doi: 10.1073/pnas.2119210119 (PMC8892508; doi:10.1073/pnas.2119210119)
Supplement: Supplementary File [file pnas.2119210119.sapp.pdf]

Supplementary Figure and Table Legends

Figure S1. Variation of tarsal bristle density within the Heteroptera. (Left) Scanning electron microscopy pictures showing the ventral part of the midleg tarsus for each investigated species. (Right) Bar plots indicating the mean tarsal bristle densities of midlegs. Terrestrial bugs have a density of tarsal bristles, which is significantly lower than that of the gerromorphan species. Within Gerromorpha, the early-diverged lineages (species that walk both on the ground and water) show a lower bristle density than derived species that specialize on open-water surface.

Figure S2. Cell division underlies embryonic leg growth in *L. dissortis*. (A-B) Late embryonic leg with cell membranes stained in red (FM4-64), nuclei in blue (DAPI), and mitotic cells in green (anti-Phospho-Histone H3). (C) Dynamics of leg growth during embryonic development with the associated number of nuclei (D) and number of dividing cells (E).

Figure S3. Quantification of leg bristle density in *G. buenoi*. RNAi-treated embryos show a significant reduction in bristle density on L1, L2 and L3 legs for the following genes: (A) *txA*, (B) *txB*, (C) *BxA*, (D) *BxB*, (E) *dodo*, (F) *GPN2*, (G) *PGMT*. YFP-treated embryos are used as negative control.

Figure S4. *In situ* hybridization showing *txA* and *txB* expression patterns. *txA* is expressed ubiquitously in late *G. buenoi* embryos, but preferentially expressed in the proximal region of the legs. At the same developmental stage, the expression of *txB* is generally stronger and covers the entire legs.

Figure S5. Phylograms of (A) Beadex homologs, (B) Simiate homologs, (C) Dodo homologs, (D) GPN-loop GTPase homologs, (E) PGMT homologs, (F) MYCBP homologs, (G) major bHLH transcription factors, obtained through maximum-likelihood analysis using the LG+Γ+I model. Bootstrap support values are indicated for each branch. Scale bar indicates the number of changes per site.

Figure S6. Comparative transcriptomic approach: methodology and candidate genes. (A) Development and post-development stages in *G. buenoi* and *M. mulsanti*. RNA-Seq libraries were made for the four following stages: katatrepsis, late embryo (katatrepsis + 46h), mid-N4, and mid-N5. (B) Trimming of the list of transcripts after read assemblies. (C) Heat map of the 1,234 differentially orthologs between *G. buenoi* and *M. mulsanti* using the number of Transcripts Per Million (TPM). (D) Sub-list of candidate genes whose bristle function is validated by RNAi.

Figure S7. Phenotypes of the forelegs and the grooming combs in RNAi-treated embryos in *G. buenoi*. The grooming combs are absent in *dodo*, *GPN2*, and *simiate* RNAi embryos. The socket cells of the grooming combs are visible, but empty, in *net* RNAi embryos RNAi. The number and/or the precise position of the grooming combs are modified in *MYCBP* and *PGMT* RNAi embryos. The scale bar indicates 20μm.

Figure S8. Functional characterization of the genes *dod* and *PGMT* in *M. mulsanti* embryos. *PGMT* RNAi knockdown resulted in a strong reduction of cuticle melanization. Bristle density and leg length are not affected. *Dod* RNAi knockdown resulted in a moderate reduction of cuticle melanization, without any apparent reduction in bristle density and leg length. The inset shows a magnified dorsal view of the abdomen. The scale bar represents 200μm.

Figure S9. Correlation between tarsus L2 bristle density and tarsus L2 length in control and knockdown individuals in *G. buenoi*. YFP: control individuals injected with ds-*YFP*.

Table S1. Accession numbers of genes markers selected for phylogenetic analyses. *5,000 Insect Genome Project (i5k); ** UniProt.

Table S2. List of PCR primers used in this study. The promoter sequence of T7RNA polymerase is indicated in bold.

Table S3. Summary of maternal RNAi efficiency in *G. buenoi* and *M. mulsanti*.

Figure S1

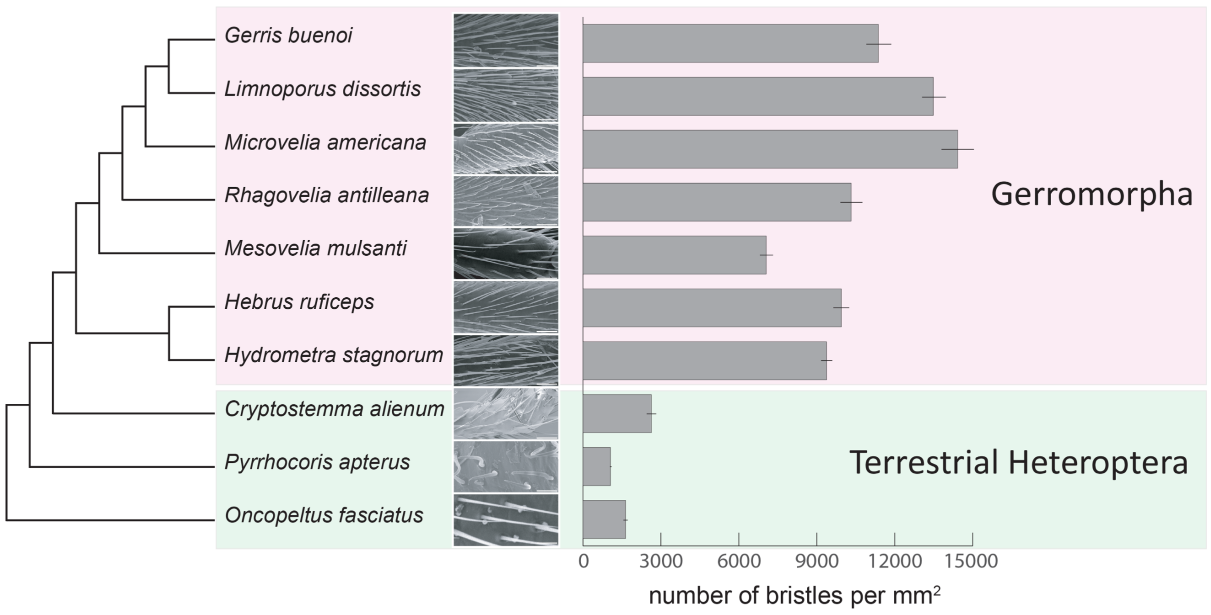
Gerris buenoi
Limnoporus dissortis
Microvelia americana
Rhagovelia antilleana
Mesovelia mulsanti
Hebrus ruficeps
Hydrometra stagnorum
Cryptostemma alienum
Pyrrhocoris apterus
Oncopeltus fasciatus
Gerromorpha
Terrestrial Heteroptera
0
3000
6000
9000
12000
15000
number of bristles per mm2

Figure S2

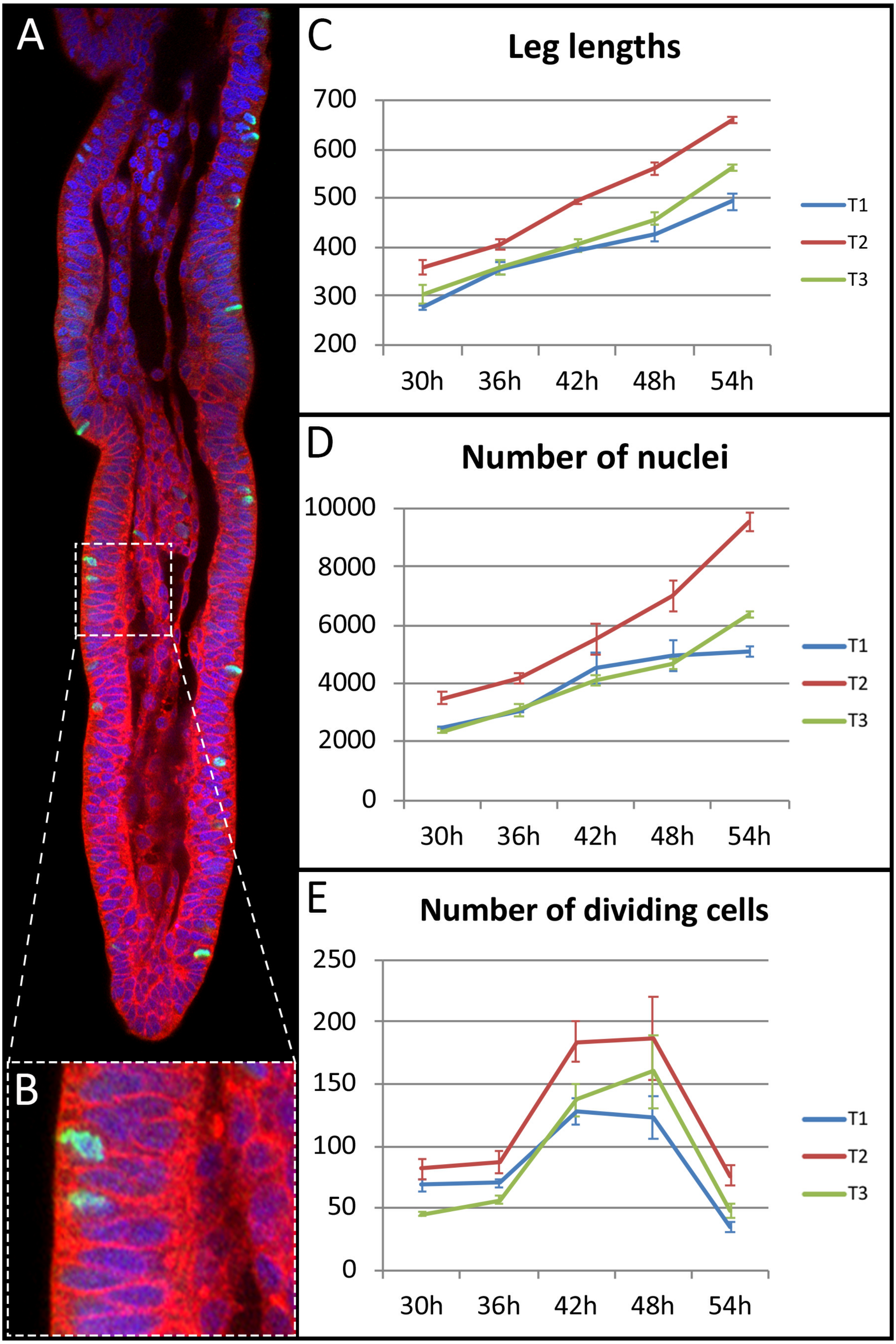
A
B
C
Leg lengths
700
600
500
400
300
200
30h
36h
42h
48h
54h
T1
T2
T3
D
Number of nuclei
10000
8000
6000
4000
2000
0
30h
36h
42h
48h
54h
T1
T2
T3
E
Number of dividing cells
250
200
150
100
50
0
30h
36h
42h
48h
54h
T1
T2
T3

Figure S3

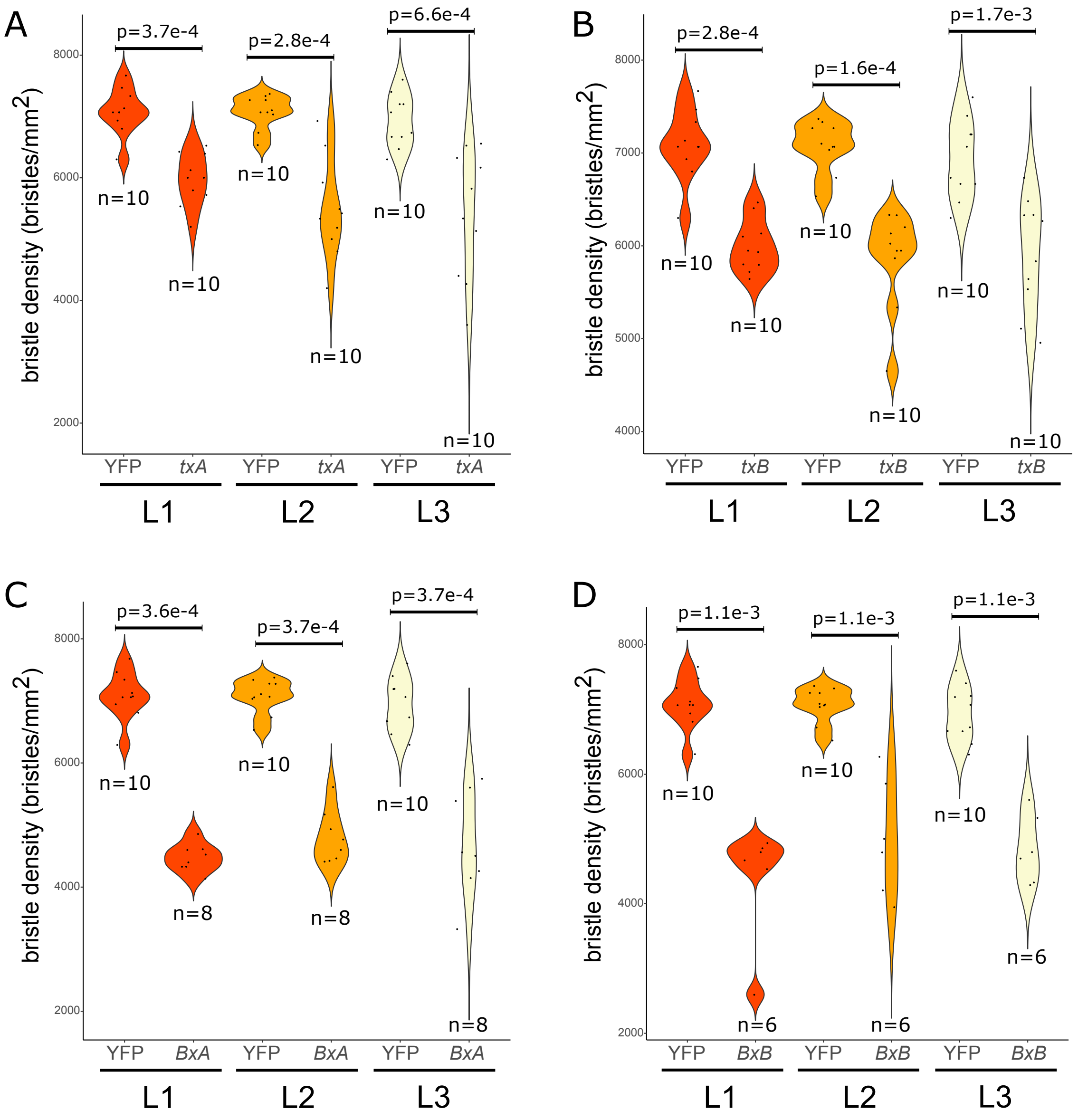

A
B
C
D
bristle density (bristles/mm2)
p=3.7e-4
p=2.8e-4
p=6.6e-4
p=1.7e-3
p=1.6e-4
p=3.6e-4
p=3.7e-4
p=1.1e-3
n=10
n=8
n=6
YFP
txA
txB
BxA
BxB
L1
L2
L3

Figure S3 (continued)

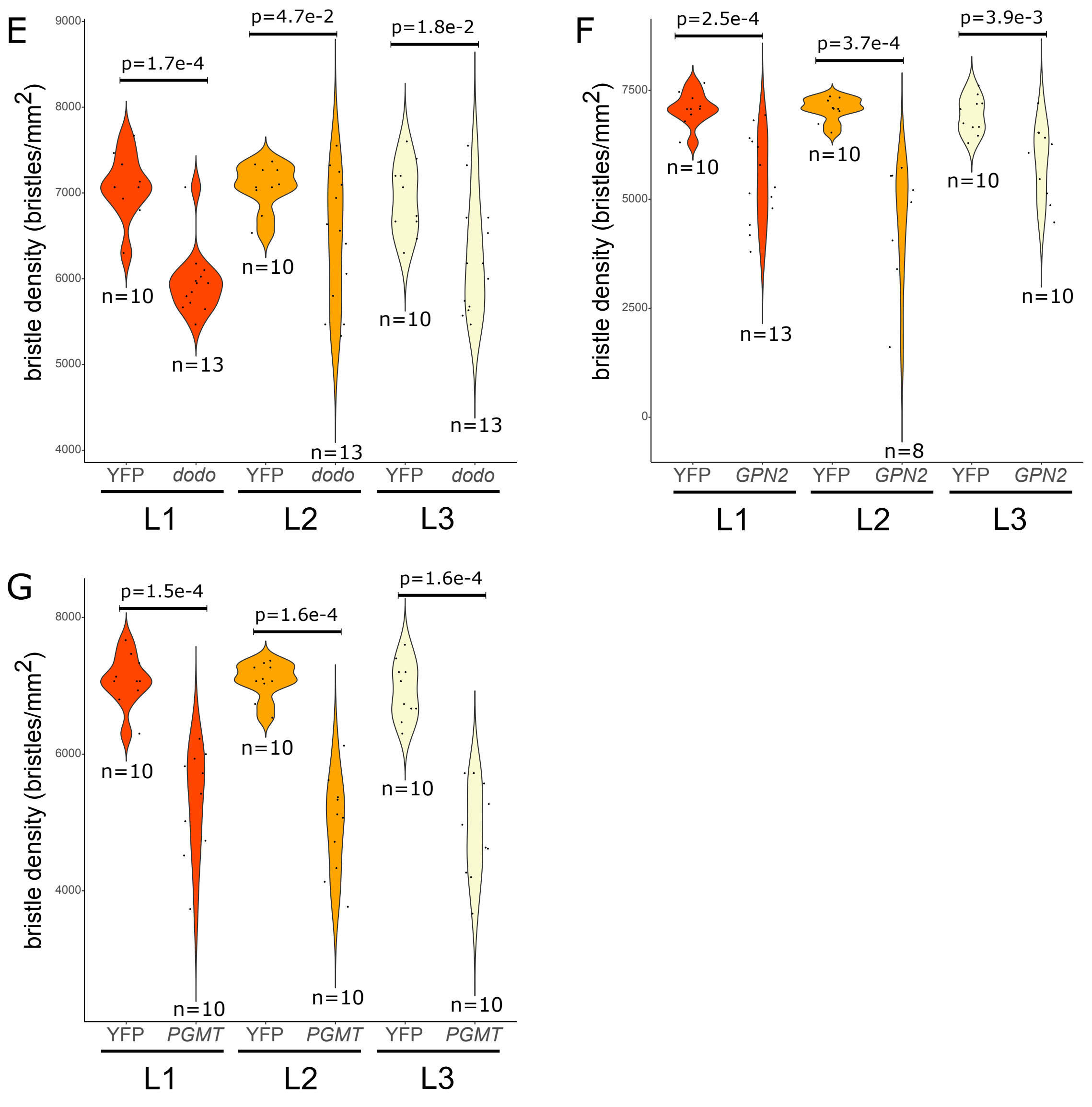

Figure S4

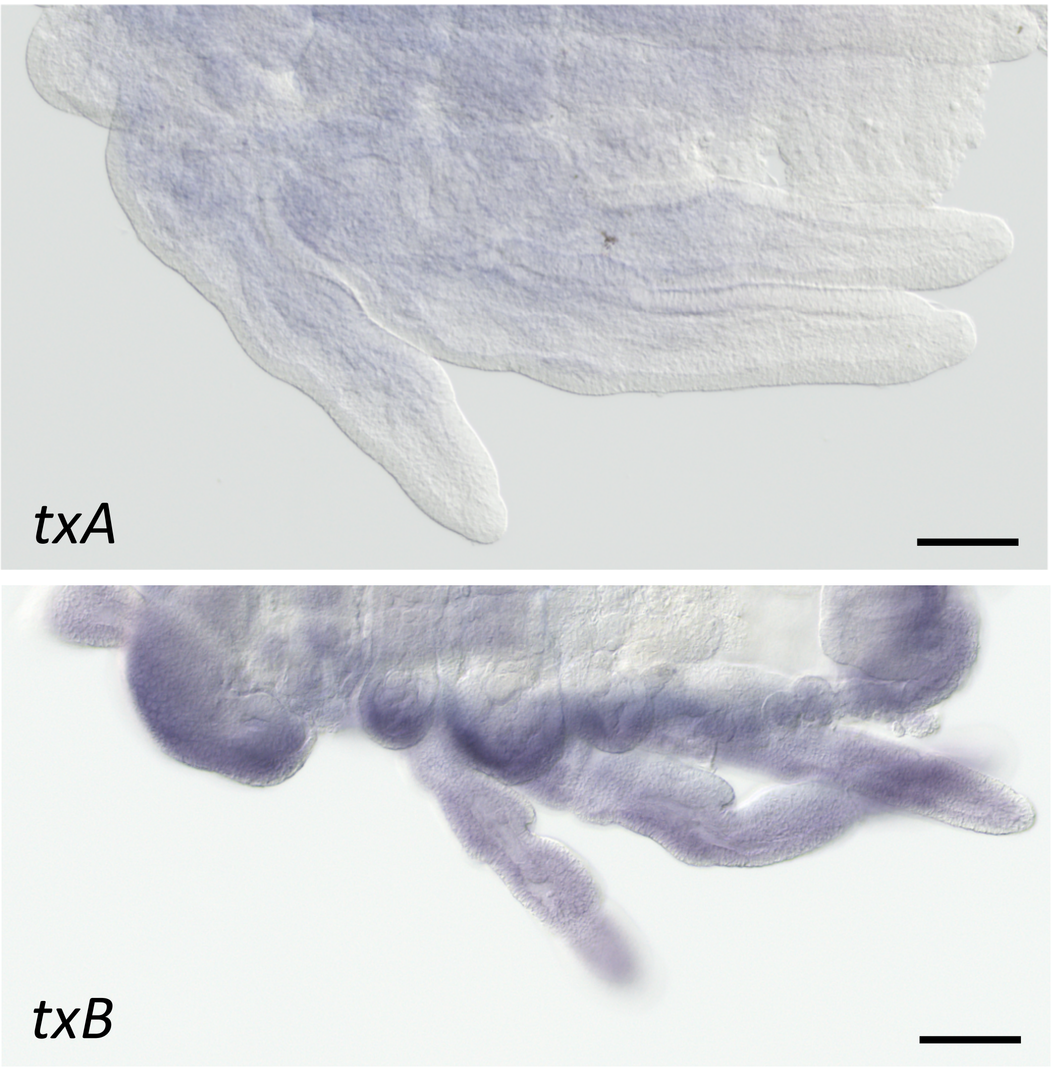

Figure S5A

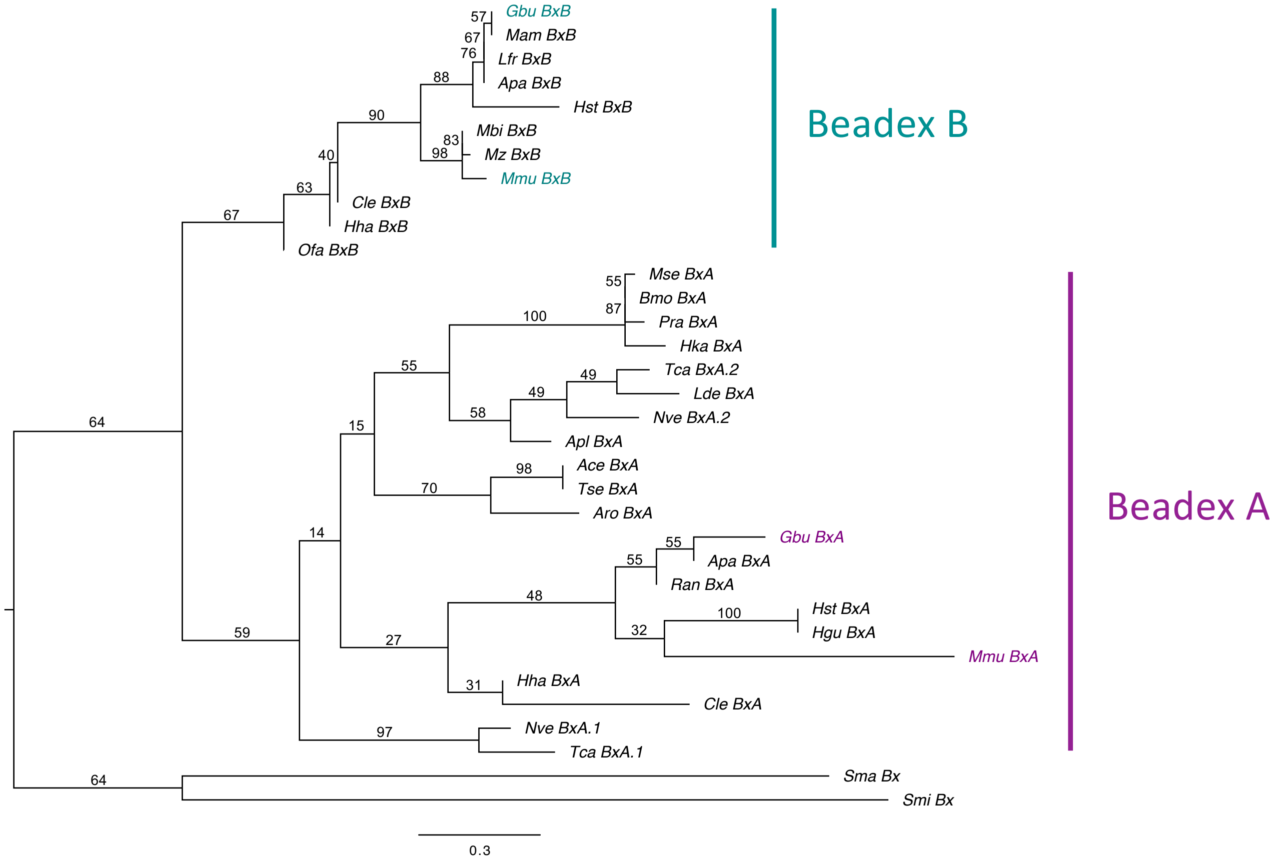

Gbu BxB
Mam BxB
Lfr BxB
Apa BxB
Hst BxB
Mbi BxB
Mz BxB
Mmu BxB
Cle BxB
Hha BxB
Ofa BxB
Beadex B
Mse BxA
Bmo BxA
Pra BxA
Hka BxA
Tca BxA.2
Lde BxA
Nve BxA.2
Apl BxA
Ace BxA
Tse BxA
Aro BxA
Gbu BxA
Apa BxA
Ran BxA
Hst BxA
Hgu BxA
Mmu BxA
Hha BxA
Cle BxA
Nve BxA.1
Tca BxA.1
Sma Bx
Smi Bx
Beadex A
0.3

Figure S5B

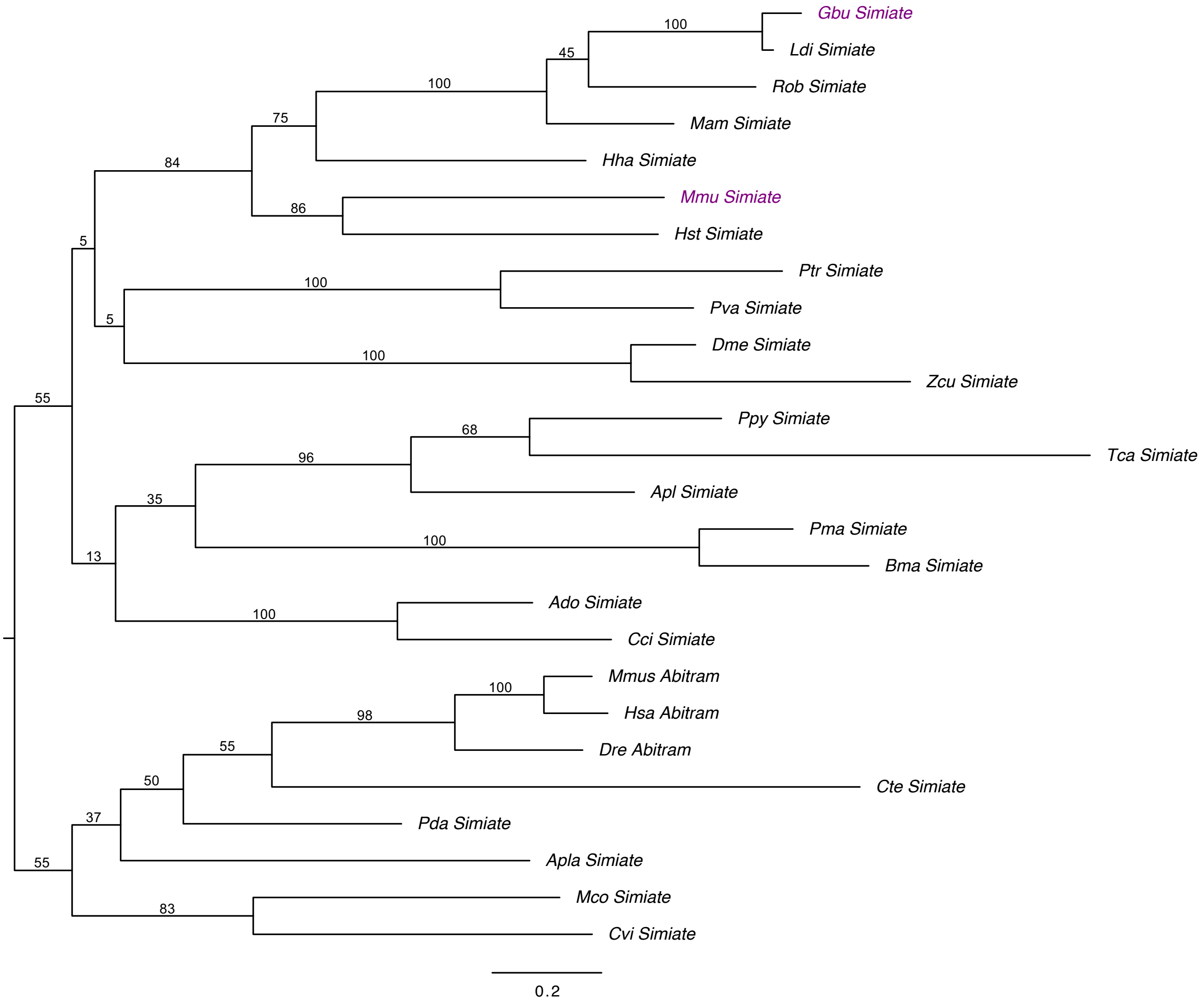
Gbu Simiate
Ldi Simiate
Rob Simiate
Mam Simiate
Hha Simiate
Mmu Simiate
Hst Simiate
Ptr Simiate
Pva Simiate
Dme Simiate
Zcu Simiate
Ppy Simiate
Tca Simiate
Apl Simiate
Pma Simiate
Bma Simiate
Ado Simiate
Cci Simiate
Mmus Abitram
Hsa Abitram
Dre Abitram
Cte Simiate
Pda Simiate
Apla Simiate
Mco Simiate
Cvi Simiate
100
45
100
75
84
86
5
100
5
100
55
68
96
35
100
13
100
100
98
55
50
37
55
83
0.2

Figure S5C

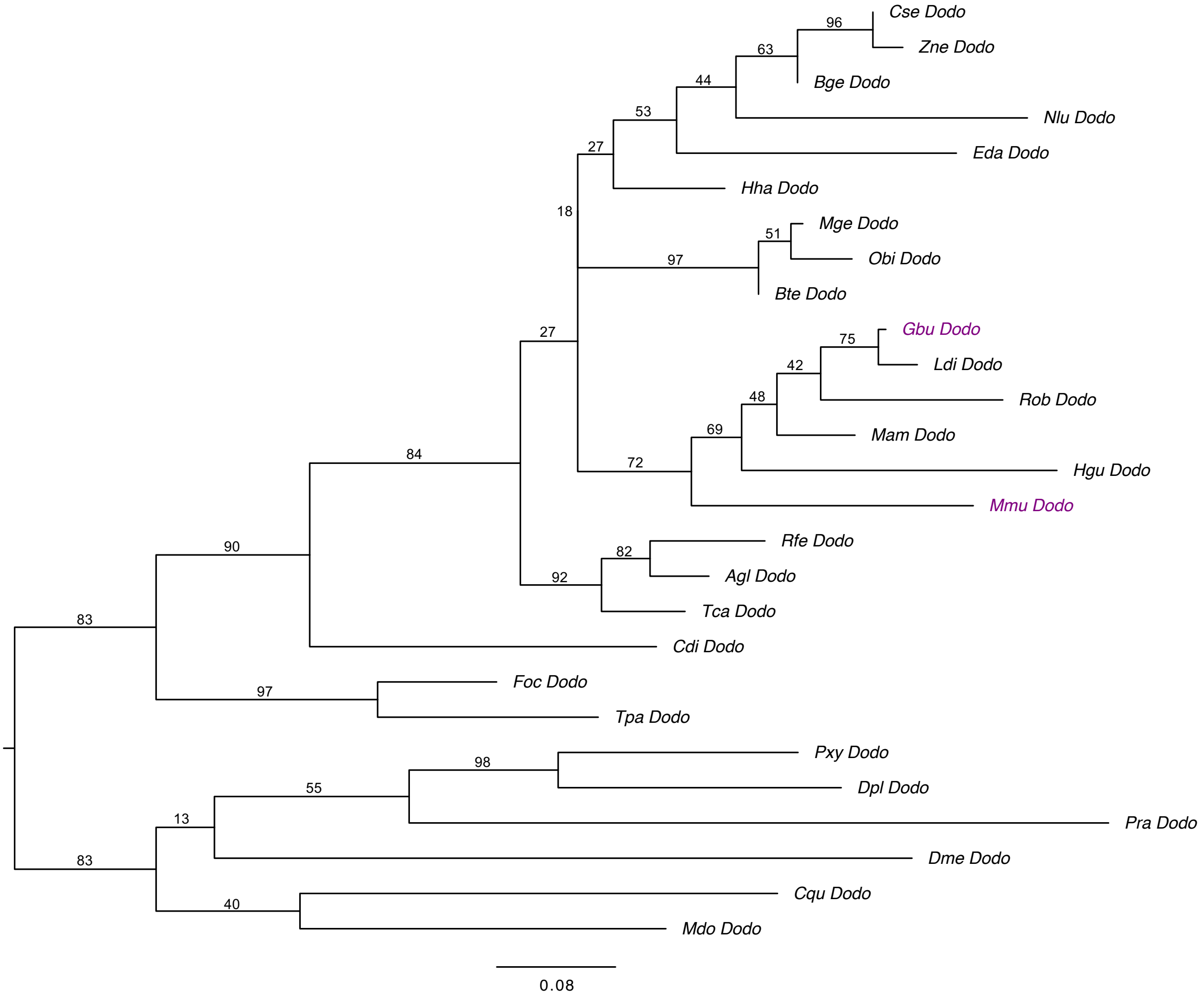

Cse Dodo
Zne Dodo
Bge Dodo
Nlu Dodo
Eda Dodo
Hha Dodo
Mge Dodo
Obi Dodo
Bte Dodo
Gbu Dodo
Ldi Dodo
Rob Dodo
Mam Dodo
Hgu Dodo
Mmu Dodo
Rfe Dodo
Agl Dodo
Tca Dodo
Cdi Dodo
Foc Dodo
Tpa Dodo
Pxy Dodo
Dpl Dodo
Pra Dodo
Dme Dodo
Cqu Dodo
Mdo Dodo
96
63
44
53
27
18
51
97
27
75
42
48
69
72
84
82
92
90
83
97
98
55
13
83
40
0.08

Figure S5D

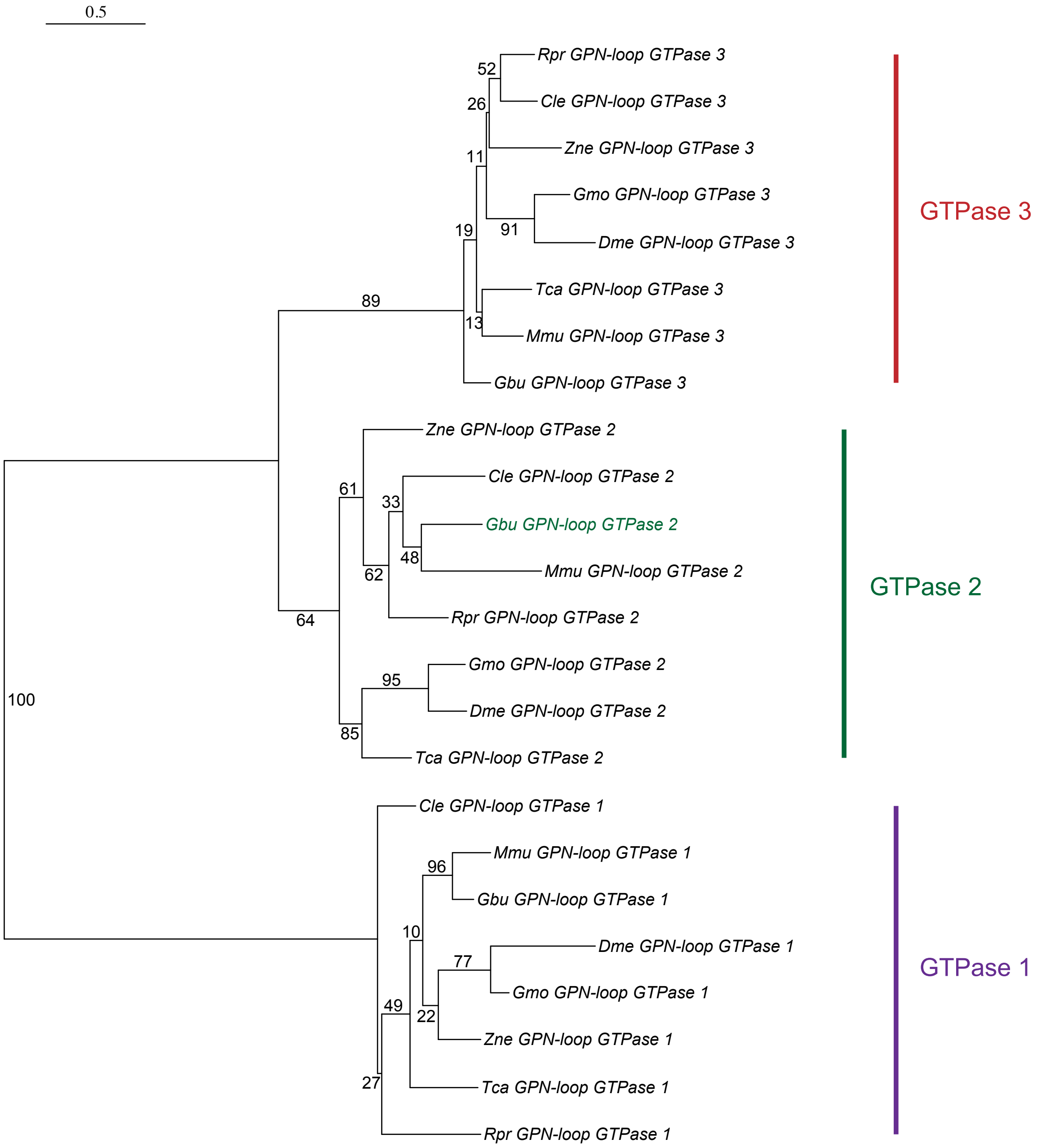
0.5
Rpr GPN-loop GTPase 3
Cle GPN-loop GTPase 3
Zne GPN-loop GTPase 3
Gmo GPN-loop GTPase 3
Dme GPN-loop GTPase 3
Tca GPN-loop GTPase 3
Mmu GPN-loop GTPase 3
Gbu GPN-loop GTPase 3
Zne GPN-loop GTPase 2
Cle GPN-loop GTPase 2
Gbu GPN-loop GTPase 2
Mmu GPN-loop GTPase 2
Rpr GPN-loop GTPase 2
Gmo GPN-loop GTPase 2
Dme GPN-loop GTPase 2
Tca GPN-loop GTPase 2
Cle GPN-loop GTPase 1
Mmu GPN-loop GTPase 1
Gbu GPN-loop GTPase 1
Dme GPN-loop GTPase 1
Gmo GPN-loop GTPase 1
Zne GPN-loop GTPase 1
Tca GPN-loop GTPase 1
Rpr GPN-loop GTPase 1
GTPase 3
GTPase 2
GTPase 1
52
26
11
19
91
89
13
61
33
48
62
64
95
85
100
96
10
77
49
22
27

Figure S5E

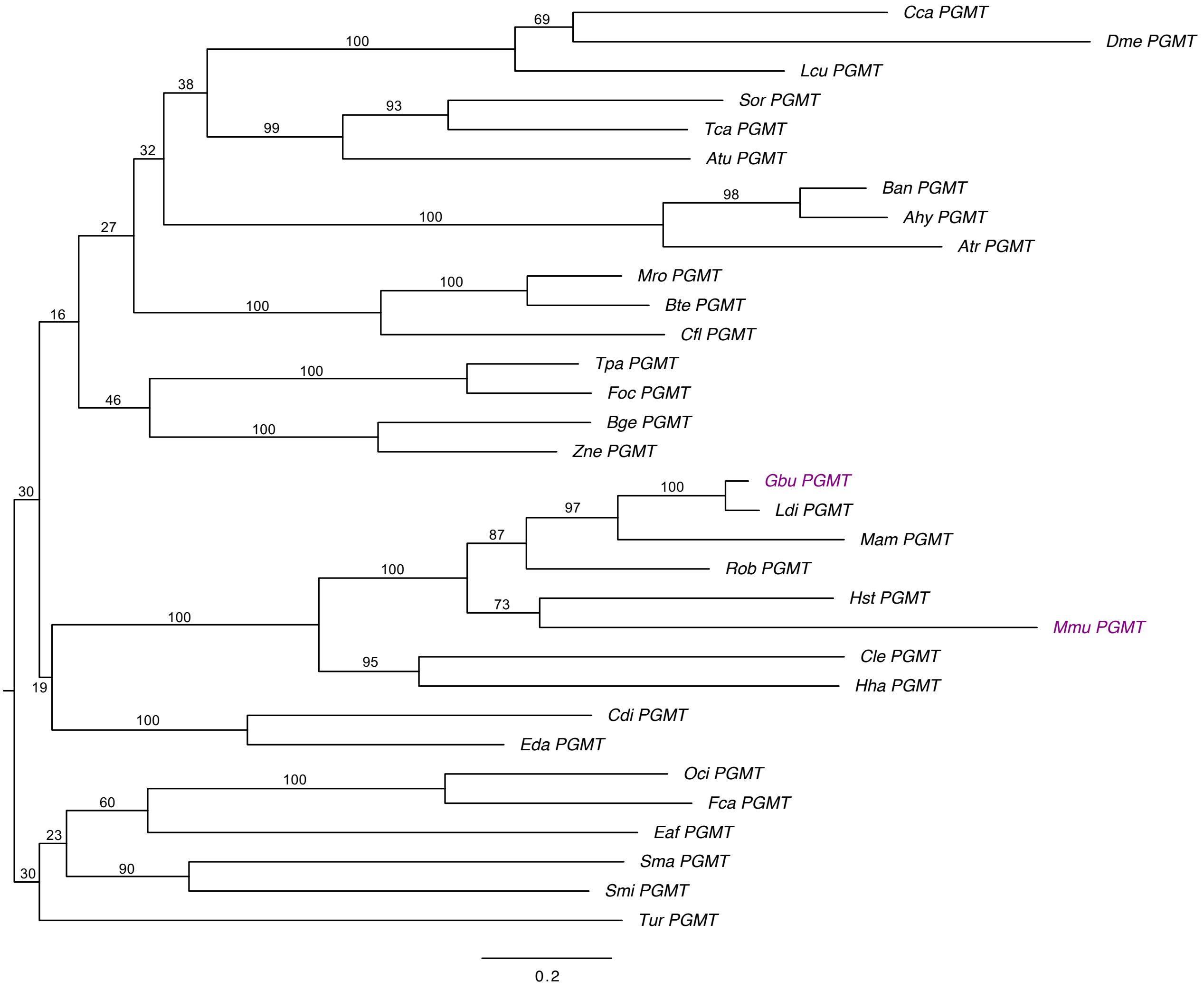
Cca PGMT
Dme PGMT
Lcu PGMT
Sor PGMT
Tca PGMT
Atu PGMT
Ban PGMT
Ahy PGMT
Atr PGMT
Mro PGMT
Bte PGMT
Cfl PGMT
Tpa PGMT
Foc PGMT
Bge PGMT
Zne PGMT
Gbu PGMT
Ldi PGMT
Mam PGMT
Rob PGMT
Hst PGMT
Mmu PGMT
Cle PGMT
Hha PGMT
Cdi PGMT
Eda PGMT
Oci PGMT
Fca PGMT
Eaf PGMT
Sma PGMT
Smi PGMT
Tur PGMT
69
100
38
93
99
32
98
100
27
100
100
16
100
46
100
100
30
97
87
100
73
100
95
19
100
100
60
23
90
30
0.2

Figure S5F

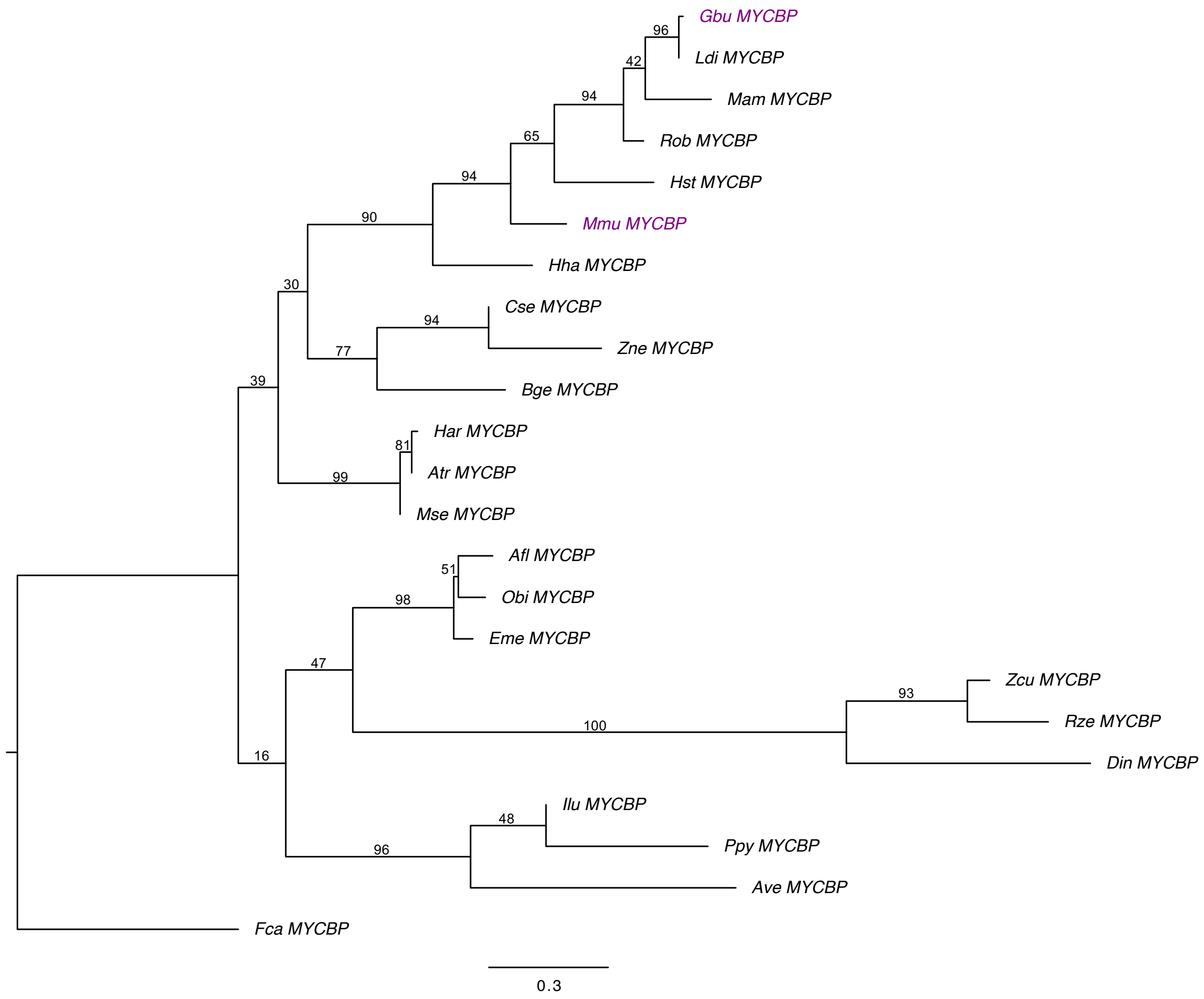
Gbu MYCBP
Ldi MYCBP
Mam MYCBP
Rob MYCBP
Hst MYCBP
Mmu MYCBP
Hha MYCBP
Cse MYCBP
Zne MYCBP
Bge MYCBP
Har MYCBP
Atr MYCBP
Mse MYCBP
Afl MYCBP
Obi MYCBP
Eme MYCBP
Zcu MYCBP
Rze MYCBP
Din MYCBP
Ilu MYCBP
Ppy MYCBP
Ave MYCBP
Fca MYCBP
96
42
94
65
94
90
30
94
77
39
81
99
51
98
47
93
100
16
48
96
0.3

Figure S5G

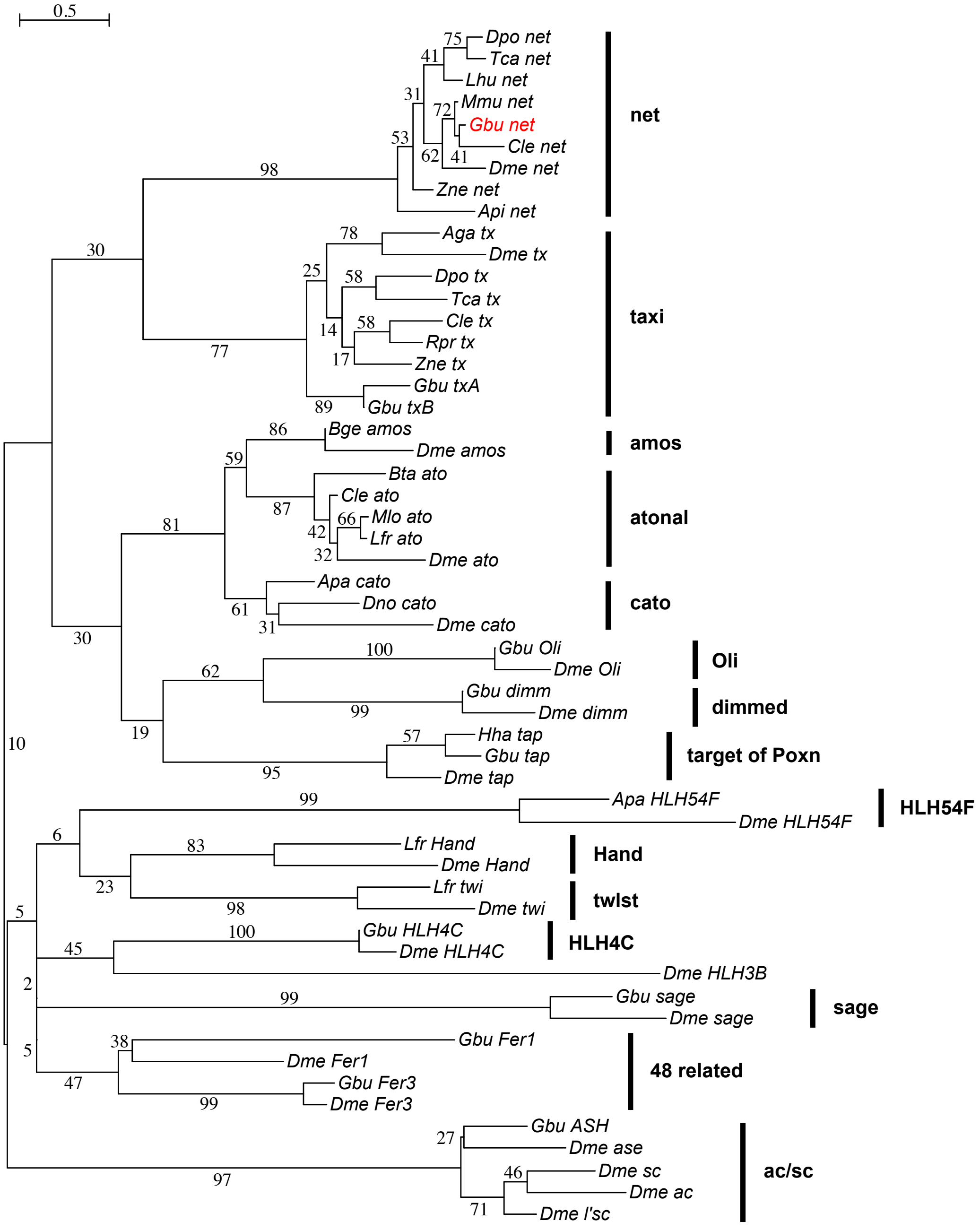
0.5
Dpo net
Tca net
Lhu net
Mmu net
Gbu net
Cle net
Dme net
Zne net
Api net
Aga tx
Dme tx
Dpo tx
Tca tx
Cle tx
Rpr tx
Zne tx
Gbu txA
Gbu txB
Bge amos
Dme amos
Bta ato
Cle ato
Mlo ato
Lfr ato
Dme ato
Apa cato
Dno cato
Dme cato
Gbu Oli
Dme Oli
Gbu dimm
Dme dimm
Hha tap
Gbu tap
Dme tap
Apa HLH54F
Dme HLH54F
Lfr Hand
Dme Hand
Lfr twi
Dme twi
Gbu HLH4C
Dme HLH4C
Dme HLH3B
Gbu sage
Dme sage
Gbu Fer1
Dme Fer1
Gbu Fer3
Dme Fer3
Gbu ASH
Dme ase
Dme sc
Dme ac
Dme l'sc
net
taxi
amos
atonal
cato
Oli
dimmed
target of Poxn
HLH54F
Hand
twlst
HLH4C
sage
48 related
ac/sc

Figure S6

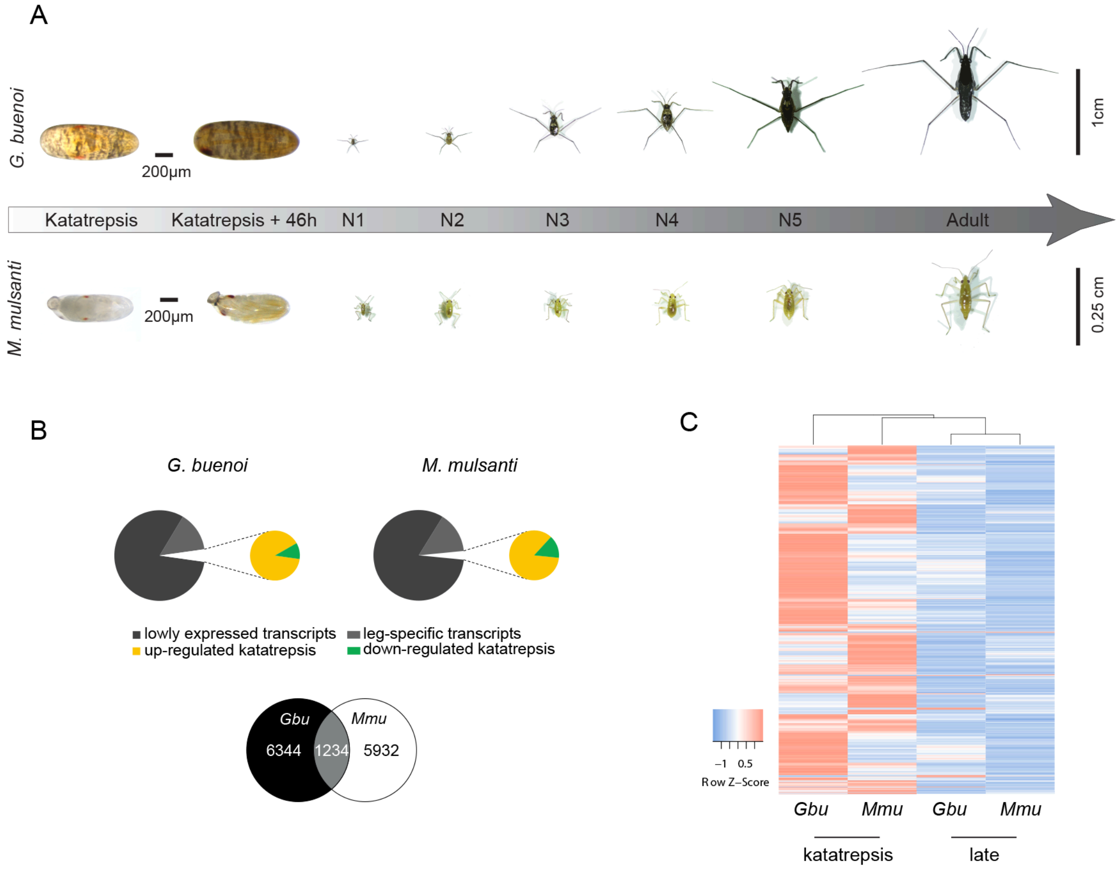

D

Gene name	GO term description	fold change		p-value		nymphal expression	
		G. buenoi	*M. mulsanti*	*G. buenoi*	*M. mulsanti*	*G. buenoi*	*M. mulsanti*
dodo	peptidyl-prolyl cis-trans isomerase	-14.99	-3.45	4.5E-08	0.026	+	-
GPN2	GTPase	-2.05	-2.91	0.001	3.3E-04	+	-
MYCBP	kinase	-2.59	-5.16	2.2E-04	1.1E-06	+	-
net	DNA-binding transcription factor	-4.12	-14.98	0.001	6.3E-04	+	-
protein-glutamate O-methyltransferase	protein carboxyl O-methyltransferase	-5.43	-3.48	0.0004	0.01	+	-
Simiate	actin filament/monomer binding	-4.21	-8.65	3.2E-08	6.26E-06	+	-

Figure S7

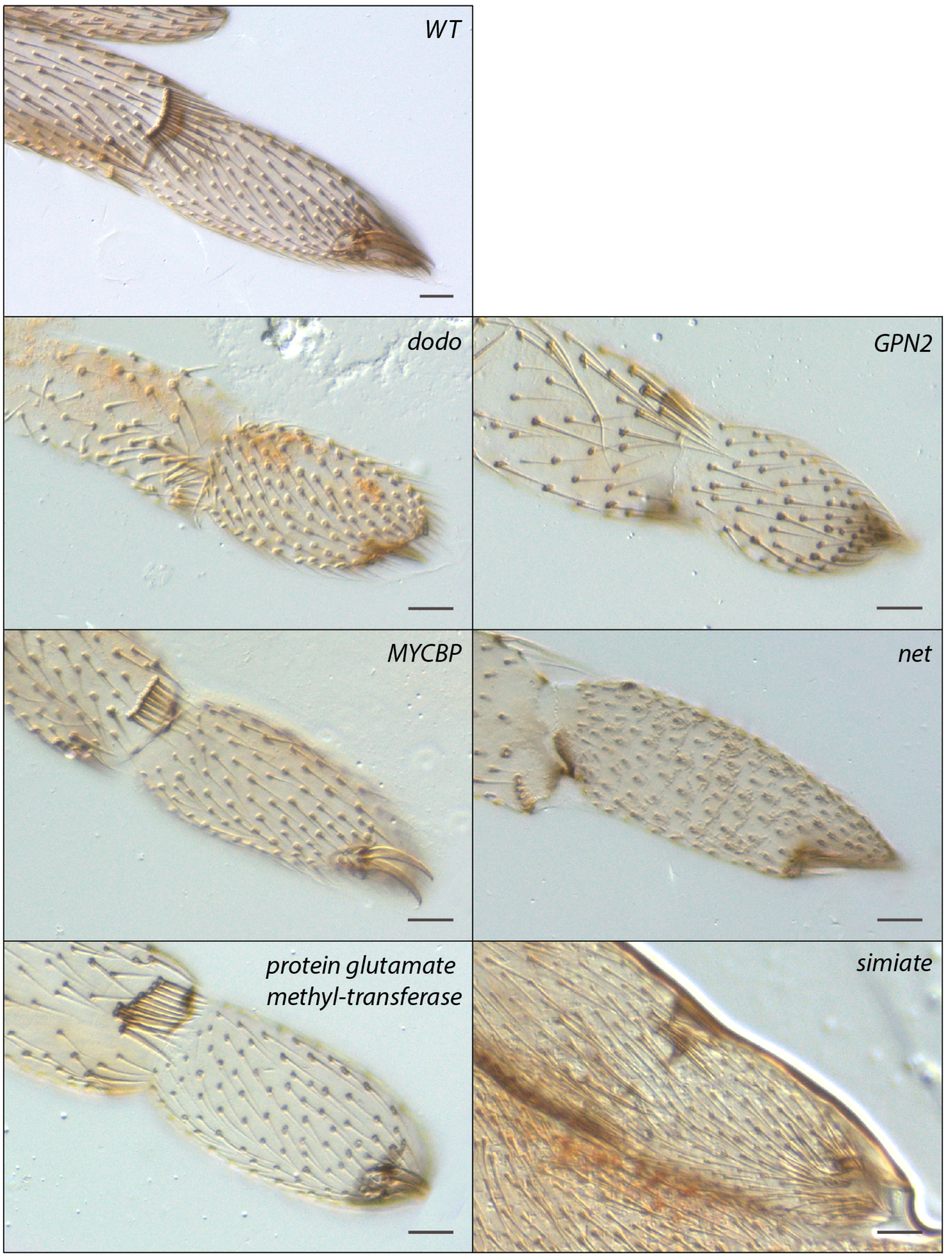
WT
dodo
GPN2
MYCBP
net
protein glutamate
methyl-transferase
simiate

Figure S8

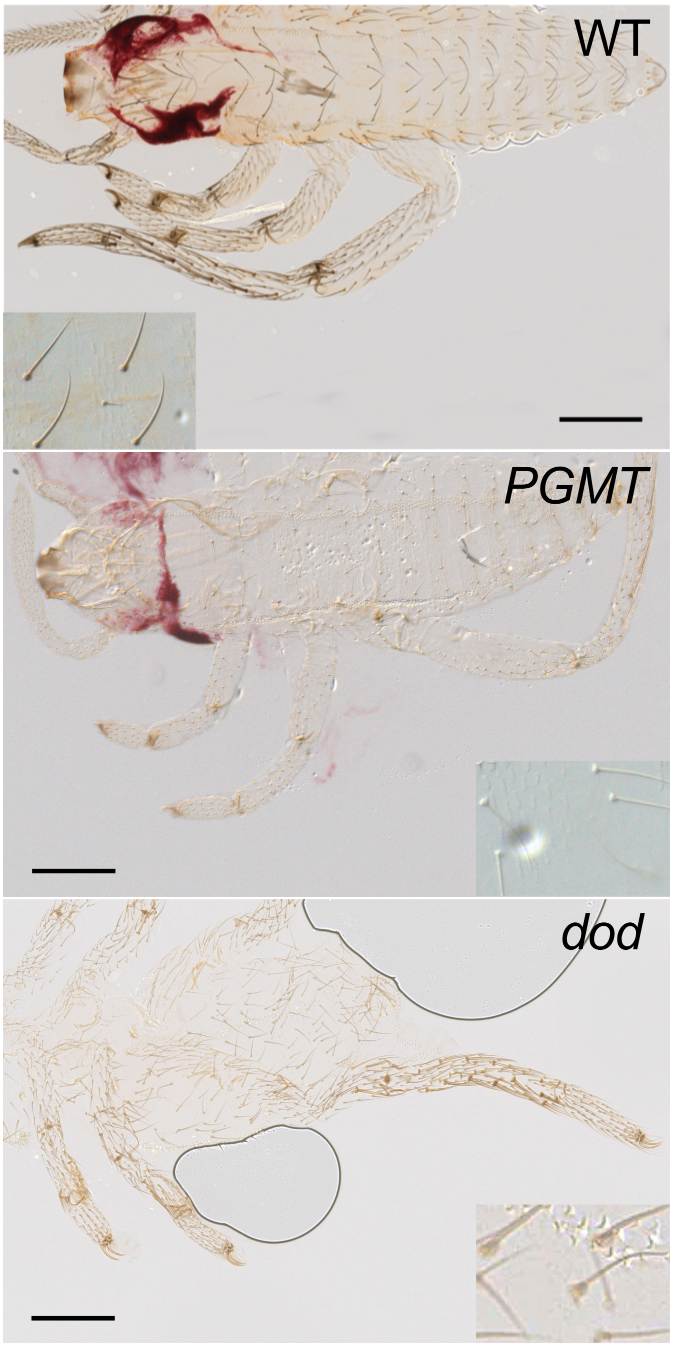

Figure S9

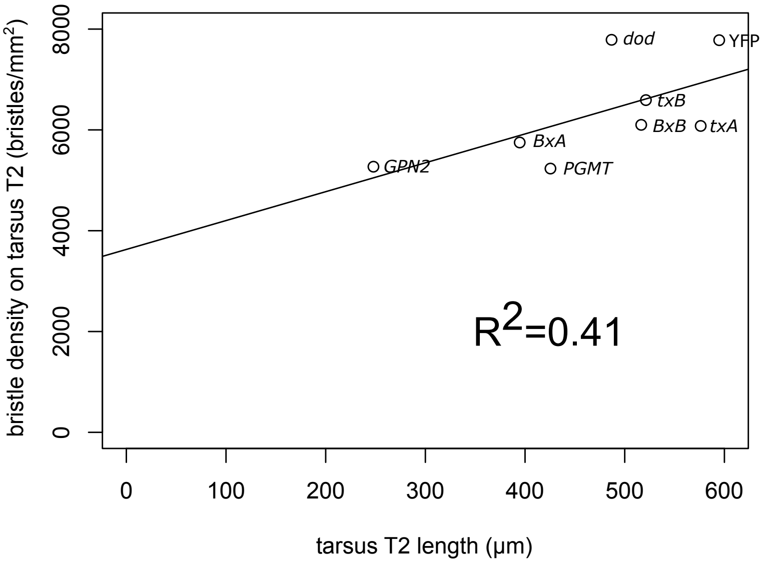

bristle density on tarsus T2 (bristles/mm2)
8000
6000
4000
2000
0
0
100
200
300
400
500
600
tarsus T2 length (µm)
dod
YFP
txB
BxB
txA
BxA
GPN2
PGMT
R2=0.41

Table S1

gene	species	accession
ac	*Drosophila melanogaster*	NM057476
amos	*Blattella germanica*	BGER020783
	Drosophila melanogaster	AF166113
ase	*Drosophila melanogaster*	NM057346
ASH	*Gerris buenoi*	LT986682
ato	*Bemisia tabaci*	XM019053204
	Cimex lectularius	XM014407032
	Drosophila melanogaster	NM169213
	Limnogonus franciscanus	LS991571
	Microvelia longipes	LS991570
bx	*Stegodyphus mimosarum*	A0A087TTW7**
	Strigamia maritima	T1JEY1**
bx (clade A)	*Agrilus planipennis*	XP018330217
	Aquarius paludum	OK267162
	Athalia rosae	AROS010003*
	Atta cephalotes	XM012208555
	Bombyx mori	NM001046789
	Cimex lectularius	CLEC000313*
	Gerris buenoi	LR027888
	Halyomorpha halys	XM014433466
	Hydrometra guianana	OK267164
	Hydrometra stagnorum	OK267165
	Hyposmocoma kahamanoa	XM026470620
	Leptinotarsa decemlineata	XM023156640
	Manduca sexta	JH668393
	Mesovelia mulsanti	LR027890
	Nicrophorus vespilloides (1)	XM017917752
	Nicrophorus vespilloides (2)	XM017919269
	Pieris rapae	XM022260420
	Rhagovelia antilleana	OK267166
	Trachymyrmex septentrionalis	XM018493165
	Tribolium castaneum (1)	XM008194456
	Tribolium castaneum (2)	XM008193709
bx (clade B)	*Aquarius paludum*	OK267163
	Cimex lectularius	CLEC002481*
	Gerris buenoi	LR027889
	Halyomorpha halys	XM024362008
	Hydrometra stagnorum	OK267167
	Limnogonus franciscanus	OK267168
	Mesovelia mulsanti	LR027891
	Mesovelia zeteki	OK267169
	Microvelia americana	OK267170
	Oncopeltus fasciatus	OFAS004517*
cato	*Aquarius paludum*	LS991572
	Diuraphis noxia	XM015520651
	Drosophila melanogaster	NM057996
dimm	*Drosophila melanogaster*	NM078887
	Gerris buenoi	LS991573
dod	*Anoplophora glabripennis*	XP018567701
	Blattella germanica	PSN41302
	Bombus terrestris	XP003397670
	Cloeon dipterum	CAB3384378

gene	species	accession
dod	*Cryptotermes secundus*	XP023727191
	Culex quinquefasciatus	EDS41533
	Danaus plexippus	XP032527468
	Drosophila melanogaster	NP523428
	Ephemera danica	KAF4517758
	Frankliniella occidentalis	XP026285393
	Gerris buenoi	LR027884
	Halyomorpha halys	XP014293033
	Hydrometra guianana	MZ712573
	Limnoporus dissortis	MZ712574
	Megalopta genalis	XP033325830
	Mesovelia mulsanti	LR027985
	Microvelia americana	MZ712575
	Musca domestica	XP005184062
	Nilaparvata lugens	XP022191655
	Ooceraea biroi	XP011333802
	Pieris rapae	XP022124847
	Plutella xylostella	XP011568532
	Rhagovelia obesa	MZ712576
	Rhynchophorus ferrugineus	KAF7271050
	Tribolium castaneum	XP008191346
	Thrips palmi	XP034255881
	Zootermopsis nevadensis	XP021921821
Fer1	*Drosophila melanogaster*	NM206455
	Gerris buenoi	LS991614
Fer3	*Drosophila melanogaster*	NM079598
	Gerris buenoi	LS991613
GPN1	*Cimex lectularius*	XP014255556
	Drosophila melanogaster	NP569872
	Gerris buenoi	LS992066
	Glossina morsitans	A0A1B0FHM2**
	Mesovelia mulsanti	LS992067
	Rhodnius prolixus	T1I4A5**
	Tribolium castaneum	XM961556
	Zootermopsis nevadensis	XM022066055
GPN2	*Cimex lectularius*	XP014242426
	Drosophila melanogaster	NP648641
	Gerris buenoi	LS991999
	Glossina morsitans	D3TRD9**
	Mesovelia mulsanti	LS992000
	Rhodnius prolixus	R4G7X0**
	Tribolium castaneum	XM008192775
	Zootermopsis nevadensis	XM022069484
GPN3	*Cimex lectularius*	XP014242422
	Drosophila melanogaster	NP649699
	Gerris buenoi	LS992068
	Glossina morsitans	A0A1B0FB01**
	Mesovelia mulsanti	LS992069
	Rhodnius prolixus	R4G389**
	Tribolium castaneum	XM001813245
	Zootermopsis nevadensis	XM022066058
Hand	*Drosophila melanogaster*	NM135526

Table S1 (continued)

gene	species	accession
Hand	*Limnogonus franciscanus*	LS991615
HLH3B	*Drosophila melanogaster*	NM080316
HLH4C	*Drosophila melanogaster*	NM057641
	Gerris buenoi	LS991618
HLH54F	*Aquarius paludum*	LS991616
	Drosophila melanogaster	NM057954
l'sc	*Drosophila melanogaster*	NM057275
MYCBP	*Amyelois transitella*	XM013336418
	Apis florea	XM003689737
	Asbolus verrucosus	RZC39939
	Blattella germanica	BGER007760*
	Cryptotermes secundus	XM033755697
	Drosophila innubila	XM034629556
	Eufriesea mexicana	XM017898227
	Folsomia candida	XM022109713
	Gerris buenoi	LR027885
	Halyomorpha halys	XM014426537
	Helicoverpa armigera	XM021326018
	Hydrometra stagnorum	MZ700332
	Ignelater luminosus	KAF2901057
	Limnoporus dissortis	MZ700333
	Manduca sexta	XM030179595
	Mesovelia mulsanti	LR027983
	Microvelia americana	MZ700334
	Osmia bicornis	XM029195041
	Photinus pyralis	XM031495967
	Rhagoletis zephyria	XM017631213
	Rhagovelia obesa	MZ700335
	Zeugodacus cucurbitae	XM011181021
	Zootermopsis nevadensis	XM022087027
net	*Acyrthosiphon pisum*	XM003245011
	Cimex lectularius	XP014241393
	Dendroctonus ponderosae	XP019760334
	Drosophila melanogaster	NM001272860
	Gerris buenoi	LS991600
	Linepithema humile	XM012360836
	Mesovelia mulsanti	LS991601
	Tribolium castaneum	XM962827
	Zootermopsis nevadensis	XM022067818
Oli	*Drosophila melanogaster*	NM001201902
	Gerris buenoi	LS991599
PGMT	*Aethina tumida*	XP019866465
	Amyelois transitella	XP013187808
	Bicyclus anynana	XP023941503
	Blattella germanica	PSN30855
	Bombus terrestris	XP003393558
	Ceratitis capitata	XP004534806
	Cimex lectularius	XP014251732
	Cloeon dipterum	CAB3383107
	Copidosoma floridanum	XP014204987
	Drosophila melanogaster	NP611635
	Ephemera danica	KAF4519307
PGMT	*Eurytemora affinis*	XP023321814
	Folsomia candida	XP021946369
	Frankliniella occidentalis	XP026274839
	Gerris buenoi	LR027886
	Halyomorpha halys	XP014282217
	Hydrometra stagnorum	MZ670771
	Limnoporus dissortis	MZ670772
	Lucilia cuprina	XP023300246
	Maniola hyperantus	XP034837651
	Megachile rotundata	XP003701663
	Mesovelia mulsanti	LR027987
	Microvelia americana	MZ670773
	Orchesella cincta	ODM99004
	Rhagovelia obesa	MZ670774
	Sitophilus oryzae	XP030752702
	Stegodyphus mimosarum	KFM76512
	Strigamia maritima	SMAR015688**
	Tetranychus urticae	XP015794842
	Thrips palmi	XP034243804
	Tribolium castaneum	XP015833761
	Zootermopsis nevadensis	XP021931469
sage	*Drosophila melanogaster*	NM079563
	Gerris buenoi	LS991617
sc	*Drosophila melanogaster*	NM057455
Simiate	*Acanthaster planci*	XP022087708
	Agrilus planipennis	XP025831214
	Apis dorsata	XP006623525
	Bombyx mandarina	XP028030993
	Capitella teleta	ELU17295
	Cephus cinctus	XP015600639
	Crassostrea virginica	XP022306199
	Danio rerio	NP001038394
	Drosophila melanogaster	NP650316
	Gerris buenoi	LR027887
	Halyomorpha halys	XP014272058
	Hydrometra stagnorum	MZ665546
	Homo sapiens	NP060302
	Limnoporus dissortis	MZ665545
	Mesovelia mulsanti	LR027986
	Microvelia americana	MZ665544
	Mus musculus	NP001074889
	Mytilus coruscus	CAC5391744
	Papilio machaon	XP014364202
	Penaeus vannamei	XP027225390
	Photinus pyralis	XP031333681
	Pocillopora damicornis	XP027036310
	Portunus trituberculatus	MPC39255
	Rhagovelia obesa	MZ665543
	Tribolium castaneum	XP008191092
	Zeugodacus cucurbitae	XP011192295
tap	*Drosophila melanogaster*	NM079400
	Gerris buenoi	LS991602

Table S1 (continued)

gene	species	accession
tap	*Halyomorpha halys*	XM014421574
twi	*Drosophila melanogaster*	NM001299823
	Limnogonus franciscanus	LS991603
tx	*Anopheles gambiae*	XM310434
	Cimex lectularius	XM014384549
	Dendroctonus ponderosae	XP019766047
	Drosophila melanogaster	NP524516
	Halyomorpha halys	XM014423440
	Oncopeltus fasciatus	OFAS010384*
	Rhodnius prolixus	T1HCE8**
	Tribolium castaneum	XP008191830
	Zootermopsis nevadensis	A0A067RT06**
tx (clade A)	*Aquarius paludum*	LS991609
	Brachymetra albinervus	LS991610
	Darwinivelia polhemi	LS992142
	Gerris buenoi	LS991611
	Husseyella diffidens	LS992146
	Husseyella halophila	LS992145
	Hydrometra comata	LS992144
	Hydrometra guianana	LS992143
	Hydrometra stagnorum	LS991612
	Limnogonus franciscanus	LS991604
	Mesovelia bila	LS992147
	Mesovelia furcata	LS991605
	Mesovelia mulsanti	LS991606
	Mesoveloidea williamsi	LS991607
	Microvelia americana	LS991608
	Microvelia longipes	LS991837
	Microvelia pulchella	LS992148
	Neogerris magnus	LS992149
	Oiovelia cunucunumana	LS991838
	Paravelia bullialata	LS991839
	Paravelia conata	LS991840
	Paravelia dilatata	LS992150
	Rhagovelia amazonensis	LS992151
	Rhagovelia antilleana	LS991841
	Rhagovelia tenuipes	LS991842
tx (clade B)	*Aquarius paludum*	LS991868
	Gerris buenoi	LS991869
	Husseyella diffidens	LS992152
	Limnogonus franciscanus	LS991845
	Paravelia dilatata	LS992153
	Rhagovelia antilleana	LS991846
	Stridulivelia strigosa	LS991847
	Velia caprai	LS991848

Table S2

species	primer name	sequence 5'-3'
G. buenoi	Gbu-*beadexA*-Fwd	CAACACTCAGCACATTGCCC
	Gbu-*beadexA*-Rev	TCTCCAACGCAGAACCTGTG
	Gbu-*beadexA*-Fwd-T7	**TAATACGACTCACTATAGGGAGACCAC**CAACACTCAGCACATTGCCC
	Gbu-*beadexA*-Rev-T7	**TAATACGACTCACTATAGGGAGACCAC**TCTCCAACGCAGAACCTGTG
	Gbu-*beadexB*-Fwd	GGAACACAAGAATGCGCAGG
	Gbu-*beadexB*-Rev	ATGGCCCACGGAAAGTCATT
	Gbu-*beadexB*-Fwd-T7	**TAATACGACTCACTATAGGGAGACCAC**GGAACACAAGAATGCGCAGG
	Gbu-*beadexB*-Rev-T7	**TAATACGACTCACTATAGGGAGACCAC**ATGGCCCACGGAAAGTCATT
	Gbu-*dodo*-Fwd	TTCGGGTTGGGAGAAGCGATTAAG
	Gbu-*dodo*-Rev	CAGTCCTGAGAATGATATGCACTC
	Gbu-*dodo*-Fwd-T7	**TAATACGACTCACTATAGGGAGACCAC**TTCGGGTTGGGAGAAGCGATTAAG
	Gbu-*dodo*-Rev-T7	**TAATACGACTCACTATAGGGAGACCAC**CAGTCCTGAGAATGATATGCACTC
	Gbu-*GPN2*-Fwd	CCTGGCAGTAAAGGGATCGT
	Gbu-*GPN2*-Rev	CATTGGTCCTCCGGGTAGTG
	Gbu-*GPN2*-Fwd-T7	**TAATACGACTCACTATAGGGAGACCAC**CCTGGCAGTAAAGGGATCGT
	Gbu-*GPN2*-Rev-T7	**TAATACGACTCACTATAGGGAGACCAC**CATTGGTCCTCCGGGTAGTG
	Gbu-*PGMT*-Fwd	ATCGACTCCGAGACTCCTCC
	Gbu-*PGMT*-Rev	TTGGCCCACAGACACAACTT
	Gbu-*PGMT*-Fwd-T7	**TAATACGACTCACTATAGGGAGACCAC**ATCGACTCCGAGACTCCTCC
	Gbu-*PGMT*-Rev-T7	**TAATACGACTCACTATAGGGAGACCAC**TTGGCCCACAGACACAACTT
	Gbu-*MYCBP*-Fwd	CCTTGGCGATACTAAGCCGT
	Gbu-*MYCBP*-Rev	AGAACTGAATGCGACAGGAGA
	Gbu-*MYCBP*-Fwd-T7	**TAATACGACTCACTATAGGGAGACCAC**CCTTGGCGATACTAAGCCGT
	Gbu-*MYCBP*-Rev-T7	**TAATACGACTCACTATAGGGAGACCAC**AGAACTGAATGCGACAGGAGA
	Gbu-*net*-Fwd	TCACACCTTGACTACCACTACTCG
	Gbu-*net*-Rev	CTCTACGCTTGGTGACGACCCGTCA
	Gbu-*net*-Fwd-T7	**TAATACGACTCACTATAGGGAGACCAC**TCACACCTTGACTACCACTACTCG
	Gbu-*net*-Rev-T7	**TAATACGACTCACTATAGGGAGACCAC**CTCTACGCTTGGTGACGACCCGTCA
	Gbu-*Simiate*-Fwd	ATCCGTCATGCAAGTTAGTA
	Gbu-*Simiate*-Rev	ACATAGTTTTACCCAAGAACCA
	Gbu-*Simiate*-Fwd-T7	**TAATACGACTCACTATAGGGAGACCAC**ATCCGTCATGCAAGTTAGTA
	Gbu-*Simiate*-Rev-T7	**TAATACGACTCACTATAGGGAGACCAC**ACATAGTTTTACCCAAGAACCA
	Gbu-*taxiA*-Fwd	TGGAACTATCATGAGGAGGTCATCG
	Gbu-*taxiA*-Rev	AGGGTCTAATGAGAGGGAATCTGGA
	Gbu-*taxiA*-Fwd-T7	**TAATACGACTCACTATAGGGAGACCAC**TGGAACTATCATGAGGAGGTCATCG
	Gbu-*taxiA*-Rev-T7	**TAATACGACTCACTATAGGGAGACCAC**AGGGTCTAATGAGAGGGAATCTGGA
	Gbu-*taxiB*-Fwd	TGCGGGCAGCTTCAGGCGAGACGAA
	Gbu-*taxiB*-Rev	AGAGGAGACATCGAAGTCCTGGAAG
	Gbu-*taxiB*-Fwd-T7	**TAATACGACTCACTATAGGGAGACCAC**TGCGGGCAGCTTCAGGCGAGACGAA
	Gbu-*taxiB*-Rev-T7	**TAATACGACTCACTATAGGGAGACCAC**AGAGGAGACATCGAAGTCCTGGAAG
M. mulsanti	Mmu-*beadexA*-Fwd	GGCCCGCATAACCATTTCAA
	Mmu-*beadexA*-Rev	GGTGGAGGTGAAAGCCCAAA
	Mmu-*beadexA*-Fwd-T7	**TAATACGACTCACTATAGGGAGACCAC**GGCCCGCATAACCATTTCAA
	Mmu-*beadexA*-Rev-T7	**TAATACGACTCACTATAGGGAGACCAC**GGTGGAGGTGAAAGCCCAAA
	Mmu-*beadexB*-Fwd	AGTGCCCATGGTAGCAGTTC
	Mmu-*beadexB*-Rev	GTGAACCCGCCGAATTAACG
	Mmu-*beadexB*-Fwd-T7	**TAATACGACTCACTATAGGGAGACCAC**AGTGCCCATGGTAGCAGTTC
	Mmu-*beadexB*-Rev-T7	**TAATACGACTCACTATAGGGAGACCAC**GTGAACCCGCCGAATTAACG
	Mmu-*dodo*-Fwd	AGCTGGATGGGAAAAACGTCTCAG
	Mmu-*dodo*-Rev	CAGTGCGCATGATAATGTGTACAC
	Mmu-*dodo*-Fwd-T7	**TAATACGACTCACTATAGGGAGACCAC**AGCTGGATGGGAAAAACGTCTCAG
	Mmu-*dodo*-Rev-T7	**TAATACGACTCACTATAGGGAGACCAC**CAGTGCGCATGATAATGTGTACAC
	Mmu-*PGMT*-Fwd	GCCAGCCGTTGAAAACAAGT
	Mmu-*PGMT*-Rev	TCCGGTAATTCAGGTCCCCT
	Mmu-*PGMT*-Fwd-T7	**TAATACGACTCACTATAGGGAGACCAC**GCCAGCCGTTGAAAACAAGT
	Mmu-*PGMT*-Rev-T7	**TAATACGACTCACTATAGGGAGACCAC**TCCGGTAATTCAGGTCCCCT

Table S3

species	gene	WT	bristle and/or leg length defects	total
G. buenoi	*Beadex A*	80	37 (32%)	117
	Beadex B	78	20 (20%)	98
	dodo	155	86 (36%)	241
	GPN2	44	16 (27%)	60
	MYCBP	54	19 (26%)	73
	net	128	40 (24%)	168
	PGMT	91	19 (17%)	110
	Simiate	103	15 (13%)	118
	taxi A	52	27 (34%)	79
	taxi B	119	45 (27%)	164
	taxi A+B	46	23 (33%)	69
M. mulsanti	*Beadex A*	57	20 (26%)	77
	Beadex B	74	22 (23%)	96
	dodo	68	13 (16%)	81
	PGMT	53	9 (15%)	62
